# Climate-Resilient Bread Wheat for Arid Environments: Adaptation and Yield Performance of Diverse Genotypes Across Contrasting Growing Conditions

**DOI:** 10.3390/plants15152385

**Published:** 2026-08-03

**Authors:** Naser B. Almarri, Mohamed Mansour, Sally E. Elwakeel, Elsayed E. Elshawy, Ibrahim F. Mersal, Abdullah D. Alkhathami, Nada M. Alsofuani, Mohamed Hichem Neily, Elsayed Mansour

**Affiliations:** Seed Center and Plant Genetic Resources Bank, Ministry of Environment, Water and Agriculture (MEWA), Riyadh 14712, Saudi Arabia; momelsayed@mewa.gov.sa (M.M.); sallyelmorsy1@gmail.com (S.E.E.); elsayed.elshawy@arc.sci.eg (E.E.E.); mrsal7108@gmail.com (I.F.M.); akorganic@mewa.gov.sa (A.D.A.); nadaalsofuani@gmail.com (N.M.A.); neilyhech@gmail.com (M.H.N.)

**Keywords:** crop improvement, genetic resources, germplasm evaluation, multi-location trials, phenological development, sustainable agriculture, yield stability

## Abstract

Environmental variability poses major challenges to bread wheat production in arid regions. This study evaluated the adaptation, productivity, and grain quality of fifteen diverse bread wheat genotypes. The evaluated germplasm comprised advanced breeding lines developed by the Arab Center for the Studies of Arid Zones and Dry Lands (ACSAD), Saudi landraces, newly released cultivars, and a cultivar derived from the International Maize and Wheat Improvement Center (CIMMYT). Field experiments were conducted over two consecutive growing seasons (2022/2023 and 2023/2024) at two contrasting arid environments in Saudi Arabia. Riyadh exhibited warmer, drier conditions, with higher soil calcium carbonate content than Hail. Significant effects (*p* ≤ 0.01) of genotype, environment, and genotype-by-environment interaction were detected for all traits studied. Compared with Hail, Riyadh exhibited lower grain and biological yields, fewer spikes/m^2^, lighter grains, reduced plant height, and shorter growth duration. Riyadh-1 achieved the highest grain yield (7.54 t/ha) and biological yield (21.44 t/ha). ACS-1454, ACS-1422, ACS-1372, and Maeaa also demonstrated superior productivity and adaptation. Local landraces were characterized by late heading and maturity, whereas ACS-1454, ACS-1422, ACS-1400, and ACS-1464 exhibited early phenology across environments. LR-12 and LR-599 exhibited the highest protein and gluten contents, whereas Riyadh-1 and Yecora recorded the highest gluten index values. Multivariate analyses, including principal component analysis, hierarchical clustering, and AMMI identified Riyadh-1, ACS-1454, ACS-1422, ACS-1372 and Maeaa as promising candidates for cultivation and breeding. Furthermore, the local landraces (LR-12 and LR-599) represent valuable sources of adaptive genetic diversity and grain quality for climate-resilient wheat cultivars.

## 1. Introduction

Bread wheat (*Triticum aestivum* L.) is one of the most important cereal crops worldwide. It is a strategic crop for food security in arid and semi-arid regions [1]. Wheat provides carbohydrates, proteins, and essential minerals [2]. Sustainable wheat production is particularly critical in arid regions. The agricultural systems in these regions are exposed to environmental stresses [3]. Several environmental challenges constrain wheat production in Saudi Arabia [4]. These constraints include limited freshwater availability, high evaporative demand, and frequent thermal extremes [5]. Climate change has intensified these stresses with considerable impacts on crop productivity [6]. In particular, elevated temperatures during the reproductive stage reduce pollen viability and increase floret sterility [7]. Heat shortens the effective grain-filling period and restricts assimilate translocation [8]. Consequently, these effects reduce grain weight and final yield [9]. The changing climatic conditions intensify the need to develop resilient wheat cultivars [10].

Genotype-by-environment interaction strongly affects wheat performance [11]. Significant interactions complicate the selection process [12]. Therefore, multi-environment trials are essential for identifying broadly adapted genotypes and evaluating the agronomic stability of candidate genotypes across diverse environments [13,14]. Grain yield is the primary criterion in wheat breeding because it integrates the cumulative effects of environmental conditions, particularly temperature variation, on crop performance [15,16]. Furthermore, phenological traits are crucial for assessing stress tolerance [17]. Days to heading and days to maturity enable heat escape through earlier development [18]. Early development helps the crop avoid the stressful phases during the reproductive stage. Likewise, plant height affects the canopy architecture [19]. Yield components provide insight into which processes are affected by abiotic stress [20].

Grain quality is a key determinant of the nutritional, technological, and commercial value of bread wheat [21]. Traits such as grain protein content, wet and dry gluten, gluten index, crude fiber, fat, and ash are complex quantitative characteristics that are influenced by both genetic background and environmental conditions [22]. Temperature during grain filling and genotype-by-environment interactions can markedly affect grain composition [23]. Consequently, wheat genotypes may differ substantially in their ability to maintain desirable grain quality under contrasting environments. Simultaneous evaluation of agronomic performance and grain quality is therefore essential for identifying superior cultivars that combine high productivity with acceptable end-use quality, particularly in arid regions where environmental stresses frequently influence both yield and grain composition.

Saudi local landraces represent valuable pools of adaptive genetic diversity. The advanced breeding lines included in this study were developed by ACSAD through regional wheat improvement programs possessing potential tolerance to environmental stresses. These lines represent advanced breeding materials selected through successive cycles for agronomic performance, adaptation, and yield potential. These lines were chosen because they represent promising advanced breeding materials available for testing under Saudi Arabian conditions. Their evaluation alongside Saudi landraces, recently released Saudi cultivars, and a CIMMYT-derived cultivar provided a comprehensive framework for comparing diverse genetic resources and identifying broadly adapted, high-yielding genotypes with potential use as candidate cultivars or parental materials in future wheat improvement programs in arid environments.

Despite recent advances in wheat improvement, comparative evaluations of Saudi landraces, newly released cultivars, and advanced breeding lines across contrasting arid environments remain limited. Historically, wheat production in Saudi Arabia has relied on a relatively narrow genetic base, consisting primarily of local landraces and the widely cultivated cultivar Yecora Rojo. To broaden genetic diversity, the Saudi Seed Center established a national wheat breeding program. However, information on comparative adaptation and performance across representative production environments remains scarce. This study addresses this knowledge gap by integrating Saudi landraces, released Saudi cultivars, ACSAD advanced breeding lines, and a CIMMYT-derived cultivar into a unified multi-environment evaluation representing the wheat-growing regions of Saudi Arabia. This approach enables assessment of genotype adaptation and yield stability, while identifying superior germplasm for cultivar deployment and future breeding. Therefore, the objectives of this study were to (i) evaluate the agronomic performance and grain quality of diverse bread wheat genotypes under contrasting arid environments, (ii) quantify the effects of genotype, environment, and genotype-by-environment interaction on phenological, morphological, and yield-related traits, and (iii) identify broadly adapted, high-yielding genotypes with potential use as candidate cultivars and parental materials for wheat improvement in arid environments.

## 2. Results

### 2.1. Analysis of Variance

The combined analysis of variance revealed highly significant (*p* ≤ 0.01) effects of genotype, environment, and their interactions for all studied traits (Table 1). The significant genotypic effects indicated considerable genetic diversity among the evaluated wheat materials. Likewise, environmental effects were highly significant for all traits, indicating a strong influence of environmental conditions on phenological, morphological, and yield characteristics. Partitioning the environmental source of variation showed that both location and year significantly affected all studied traits (*p* ≤ 0.01). The magnitude of the mean squares for location was greater than that for year, indicating that differences among testing locations contributed more strongly to phenotypic variation than seasonal fluctuations between years. These findings are consistent with the pronounced climatic differences between Hail and Riyadh, particularly temperature regimes during the growing season. The warmer conditions in Riyadh likely contributed to the observed differences in crop development, growth, and productivity. The location-by-year interaction was significant for all traits except plant height and spike length. Highly significant genotype-by-environment interactions were detected for all traits except spike length. The genotype-by-location and genotype-by-year interactions were significant for most traits. However, the mean squares for genotype-by-location interaction were higher than those for genotype-by-year interaction. These results highlighted the importance of multi-location testing for identifying broadly adapted genotypes under arid conditions. The three-way interaction (genotype by location by year) was significant for all traits except spike length and days to heading. The first two interaction principal component (PCs) axes captured the largest proportion of the genotype-by-environment interaction pattern. The first two PCs accounted for most of the differential genotype responses across environments, indicating that they adequately summarized the interaction structure for phenological, morphological, and yield-related traits.

### 2.2. Main Performance

Table 2 summarizes the mean performance of the fifteen bread wheat genotypes across the two locations, Hail and Riyadh, averaged over the 2022/2023 and 2023/2024 growing seasons. Environmental differences strongly influenced genotype performance. Across all genotypes, Riyadh consistently produced lower grain yield, biological yield, plant height, spike length, number of spikes/m^2^ and thousand-grain weight than Hail. Heading and maturity occurred earlier in Riyadh, indicating accelerated crop development under the warmer environmental conditions. Among the evaluated genotypes, Riyadh-1 exhibited the highest productivity in both locations, recording grain yields of 8.06 and 7.03 t/ha and biological yields of 23.09 and 19.79 t/ha in Hail and Riyadh, respectively. The ACSAD breeding lines ACS-1454 and ACS-1422 also maintained consistently high grain and biological yields across both locations, demonstrating broad adaptation to contrasting arid environments. The released Saudi cultivar Maeaa and the breeding line ACS-1372 likewise exhibited superior productivity compared with most of the remaining genotypes. In contrast, LR-920, LR-12, ACS-1456, and ACS-1464 produced comparatively lower grain yields, particularly under the warmer Riyadh environment. Substantial variation was also observed for yield components, plant architecture, and phenological traits. Riyadh-1 produced the highest spike density, followed by Maeaa and ACS-1454, whereas LR-920 consistently recorded the fewest spikes/m^2^. Thousand-grain weight ranged from 41.6 to 47.1 g in Hail and from 36.1 to 41.4 g in Riyadh, with LR-920 and ACS-1422 producing the heaviest grains. The Saudi landrace LR-12 was the tallest genotype, reaching 125.2 cm in Hail and 110.2 cm in Riyadh, whereas Yecora consistently exhibited the shortest plants. The longest spikes were produced by ACS-1444 and ACS-1454, while Yecora and LR-920 had the shortest spikes. For phenological development, the ACSAD breeding lines, particularly ACS-1454, ACS-1422, and ACS-1400, exhibited the earliest heading and maturity across both environments, whereas the Saudi landraces LR-12, LR-599, and LR-920 maintained the longest growth duration.

**Figure 1:**
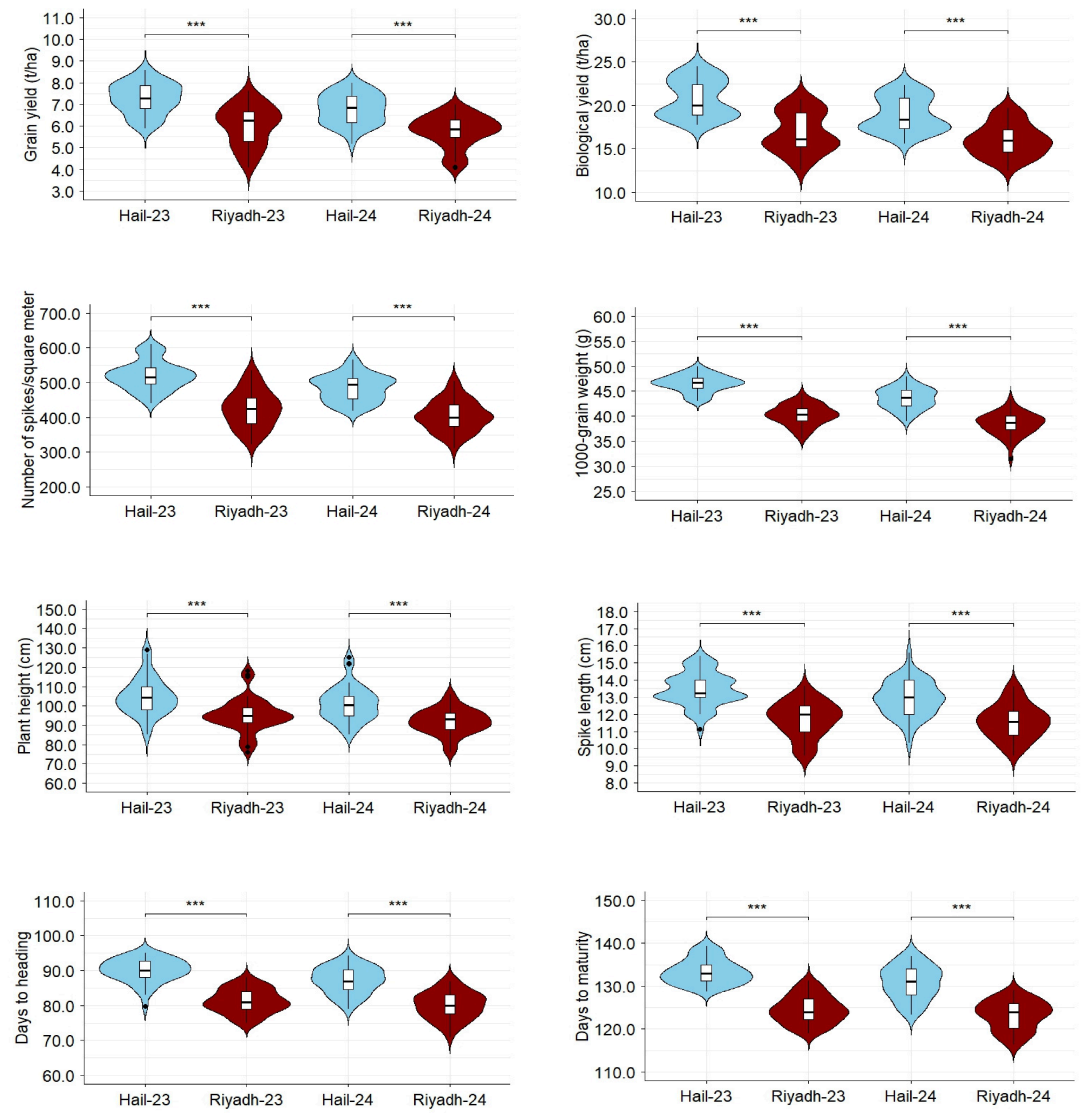
Violin plots of grain yield, biological yield, yield-contributing traits, plant stature, spike length, and phenological performance of fifteen bread wheat genotypes evaluated in Hail and Riyadh during the 2023 and 2024 growing seasons. *** indicates statistical significance at *p* < 0.001.

The violin plots in Figure 1 revealed significant differences among the four tested environments for all studied traits. Grain yield and biological yield were consistently lower and exhibited distinct distribution patterns in Riyadh-23 and Riyadh-24 environments than in Hail-23 and Hail-24. Similarly, the distributions of spike number and thousand-grain weight were shifted toward lower values in Riyadh, indicating reduced yield potential under warmer conditions. The warmer conditions in Riyadh reduced both the number of productive spikes and grain weight. Plant height and spike length were lower and had narrower distributions under Riyadh conditions. The reduction in plant stature and spike length under Riyadh conditions suggests that elevated temperatures limited stem elongation and spike growth. For the phenological traits, genotypes grown in Hail exhibited longer developmental periods than those grown in Riyadh. The warmer conditions in Riyadh accelerated phenological development and reduced the duration required to reach heading and maturity. Similar environmental effects were observed for the morphological traits.

#### 2.2.1. Grain and Biological Yields

Significant variation was observed among the evaluated wheat genotypes for grain yield across the tested environments (Table 3). Grain yield ranged from 4.243 t/ha (LR-920 in Riyadh 2023) to 8.327 t/ha (Riyadh-1 in Hail 2023). The local variety Riyadh-1 produced the highest grain yield across the environments. Also, ACS-1454, ACS-1422, Maeaa, and ACS-1372 recorded high values. In contrast, LR-920, LR-12, ACS-1456, and ACS-1464 recorded the lowest mean grain yields. In Hail 2023, Riyadh-1 achieved the highest grain yield. In addition, ACS-1454, ACS-1372, Maeaa, and ACS-1422 produced high grain yields. Conversely, LR-920, LR-12, and ACS-1456 recorded the lowest yields. In Hail 2024, Riyadh-1, ACS-1454, ACS-1444, ACS-1372, ACS-1422, and Maeaa were the highest-yielding genotypes, whereas LR-920, ACS-1464, and LR-12 exhibited lower productivity. Under Riyadh conditions, grain yield declined for all genotypes. During 2023, Riyadh-1 possessed the highest yield, followed by ACS-1422, ACS-1454, Maeaa, and ACS-1372. In contrast, LR-920, LR-12, and ACS-1456 recorded the lowest yields. In 2024, Riyadh-1 produced the highest grain yield, followed by Maeaa, ACS-1454, ACS-1372, and ACS-1422, while LR-920, LR-12, and ACS-1456 were the lowest-yielding genotypes.

Biological yield ranged from 13.14 t/ha (ACS-1456 in Riyadh 2024) to 24.13 t/ha (Riyadh-1 in Hail 2023) (Table 3). Riyadh-1 recorded the highest biological yield (21.44 t/ha), followed by ACS-1454, ACS-1422, Maeaa, and ACS-1372. In contrast, ACS-1456, LR-920, ACS-1400, Asier-1, and Yecora recorded the lowest biological yields. In Hail 2023, Riyadh-1, ACS-1422, Maeaa, ACS-1454, and ACS-1372 produced the greatest biomass accumulation. Conversely, Yecora, LR-920, and ACS-1456 recorded the lowest biological yields. In Hail 2024, Riyadh-1, ACS-1454, ACS-1372, Maeaa, and ACS-1422 were the highest-performing genotypes. Asier-1, Yecora, and LR-12 produced lower biomass. Under Riyadh conditions, ACS-1422 and Riyadh-1 recorded the highest biological yields during 2023, followed by ACS-1454 and Maeaa. ACS-1456, LR-920, and ACS-1400 exhibited the lowest values. During 2024, Riyadh-1 and ACS-1454 were superior, followed by ACS-1422 and Maeaa. ACS-1456, ACS-1400, and LR-920 recorded the lowest biomass production.

#### 2.2.2. Yield-Contributing Traits

Significant variation was observed among the evaluated wheat genotypes for the number of spikes/m^2^ across the tested environments (Table S1). The number of spikes/m^2^ ranged from 336.9 (ACS-1400 in Riyadh 2024) to 600.3 (Riyadh-1 in Hail 2023). The local variety Riyadh-1 produced the highest number of spikes/m^2^ across the environments. Also, Maeaa and ACS-1454 recorded high number of spikes/m^2^. In contrast, LR-920, ACS-1400, and LR-12 exhibited the lowest spike densities. In Hail 2023, Riyadh-1 and Maeaa recorded the highest spike counts per m^2^. Also, ACS-1454, Asier-1, and ACS-1422 possessed high number of spikes/m^2^. Conversely, LR-920, ACS-1400, and LR-12 produced the fewest spikes. In Hail 2024, Riyadh-1, Maeaa, ACS-1454, ACS-1456, and Asier-1 exhibited the highest spike densities. LR-920, ACS-1464, and ACS-1400 were the poorest performers. Under Riyadh conditions, spike density declined for all genotypes. During 2023, Riyadh-1 had the highest value. Also, Maeaa, ACS-1454, and Asier-1 recorded high values. LR-920, ACS-1400, ACS-1456, and LR-12 recorded the lowest values. During 2024, Riyadh-1 produced the highest spike density. In addition, Asier-1, ACS-1454, Maeaa, and ACS-1422 recorded high values. ACS-1400, LR-920, LR-12, and ACS-1456 exhibited the lowest spike densities.

Substantial variation was observed among genotypes for thousand-grain weight (Table S1). It ranged from 34.11 g (ACS-1372 in Riyadh 2024) to 49.18 g (LR-920 in Hail 2023). ACS-1422 and LR-920 recorded the highest overall 1000-grain weights (44.19 and 44.15 g). Riyadh-1, LR-12, ACS-1464, and ACS-1400 recorded high values. Conversely, LR-599 exhibited the lowest grain weight with an overall mean of 38.85 g. In Hail 2023, LR-920, ACS-1422, ACS-1464, Riyadh-1, and LR-12 produced the heaviest grains. LR-599 recorded the lightest grains. In Hail 2024, ACS-1444, ACS-1456, ACS-1422, LR-920, and LR-12 possessed superior grain weights. LR-599 and ACS-1372 had the lowest grain weight. Under Riyadh conditions in 2023, ACS-1422 produced the heaviest grains, followed by Riyadh-1, ACS-1454, Yecora, and ACS-1400. ACS-1456 and LR-599 recorded the lowest values. During 2024, LR-920 achieved the highest 1000-grain weight. Also, ACS-1464, ACS-1400, ACS-1454, and ACS-1422 possessed high values. ACS-1372 and LR-599 exhibited the lightest grains.

#### 2.2.3. Plant Stature and Spike Length

Significant variation was observed among the tested genotypes for plant height across the four environments (Table S2). Plant height ranged from 77.40 cm (Yecora in Riyadh 2024) to 127.48 cm (LR-12 in Hail 2023). The local landraces exhibited taller plant height than the advanced breeding lines. LR-12 was the tallest genotype followed by Maeaa and LR-920. In contrast, ACS-1464, ACS-1454, ACS-1400, and ACS-1422 were the shortest ones. Yecora recorded the shortest plant height across environments (82.51 cm). In Hail 2023, LR-12, Maeaa, and LR-920 recorded the highest plant heights. Conversely, Yecora, LR-599 and ACS-1454 were the shortest genotypes. In Hail 2024, LR-12, Maeaa, and ACS-1456 were the tallest genotypes, whereas Yecora, ACS-1464, and ACS-1454 recorded the lowest values. Under Riyadh conditions, plant height decreased for most genotypes. During 2023, LR-12 displayed the tallest plants followed by Maeaa and ACS-1372. Yecora and ACS-1464 exhibited the shortest plants (78.33–83.23 cm). During 2024, LR-12, Maeaa, ACS-1372, and ACS-1444 were the tallest ones, while Yecora, ACS-1464, and ACS-1454 were the shortest genotypes.

The evaluated genotypes exhibited significant differences for spike length (Table S2). Spike length ranged from 10.27 cm (LR-920 in Riyadh 2023) to 14.80 cm (ACS-1454 in Hail 2023). The ACSAD advanced line ACS-1454 produced the longest spikes across environments (13.65 cm). Also, ACS-1346, ACS-1444, and Riyadh-1 possessed high spike length. In contrast, Yecora, LR-920, and ACS-1464 exhibited the shortest spikes. The local landraces displayed intermediate spike lengths. In Hail 2023, ACS-1454, ACS-1444, ACS-1464, and Riyadh-1 produced the longest spikes. Yecora and LR-920 recorded the shortest spikes. In Hail 2024, ACS-1444, ACS-1454, Riyadh-1, and ACS-1346 had high spike length. Yecora and ACS-1464 recorded the shortest spikes. In Riyadh 2023, ACS-1454, ACS-1346, and ACS-1400 exhibited the highest spike length, whereas ACS-1464, LR-920, and Yecora produced the shortest spikes. During 2024, ACS-1454, ACS-1346, and ACS-1422 had longer spikes, while Yecora, ACS-1456, and ACS-1464 recorded the lowest values.

#### 2.2.4. Phenological Traits

Substantial variation was observed among the evaluated wheat genotypes for days to heading and days to physiological maturity (Table S3). Days to heading ranged from 72.63 days (ACS-1422 in Riyadh 2024) to 94.49 days (LR-12 in Hail 2023). Across environments, the local landraces LR-12, LR-599, and LR-920 exhibited the latest heading dates. In contrast, the ACSAD advanced lines ACS-1454, ACS-1422, ACS-1444, and ACS-1400 exhibited earlier heading. The local varieties displayed intermediate responses, except Maeaa, which was late under Hail conditions. The CIMMYT cultivar Yecora also exhibited relatively early heading. LR-12, LR-599, LR-920, and Maeaa exhibited the latest heading in Hail during both seasons. In contrast, ACS-1454 and Yecora were the earliest genotypes in Hail 2023, while ACS-1454, Yecora, ACS-1464, and ACS-1422 recorded the earliest heading in 2024. Under Riyadh 2023, ACS-1422, Asier-1, and ACS-1454 exhibited the earliest heading dates. LR-12 and LR-920 were the latest genotypes. In 2024, ACS-1422, ACS-1454, ACS-1400, Yecora, and Asier-1 were the earliest ones, while LR-12 and LR-920 recorded the latest heading.

Similarly, significant differences were observed among the tested genotypes for days to physiological maturity across environments (Table S3). Days to maturity ranged from 117.8 days (CS-1422 in Riyadh 2024) to 138.4 days (LR-599 in Hail 2023).

The local landraces exhibited the longest maturity duration, with LR-12, LR-599, and LR-920 consistently required more days to reach physiological maturity than the remaining genotypes. In contrast, the ACSAD advanced lines exhibited early maturity. In Hail 2023, LR-599, ACS-1346, LR-920, and LR-12 recorded the latest maturity. ACS-1454 and ACS-1400 recorded the earliest maturity. In Hail 2024, ACS-1346, LR-12, LR-599, and LR-920 exhibited the latest maturity, while ACS-1464, Riyadh-1, ACS-1454, and ACS-1400 exhibited the earliest maturity. In Riyadh 2023, Maeaa, ACS-1346, and LR-12 recorded the latest maturity. ACS-1456, Riyadh-1, ACS-1464, and ACS-1454 displayed the earliest maturity. In Riyadh 2024, Maeaa and LR-12 were the latest genotypes, while ACS-1422, Riyadh-1, ACS-1454, and ACS-1400 possessed the earliest maturity.

### 2.3. Genotypic Classification Based on Agronomic Performance Across Environments

**Figure 2:**
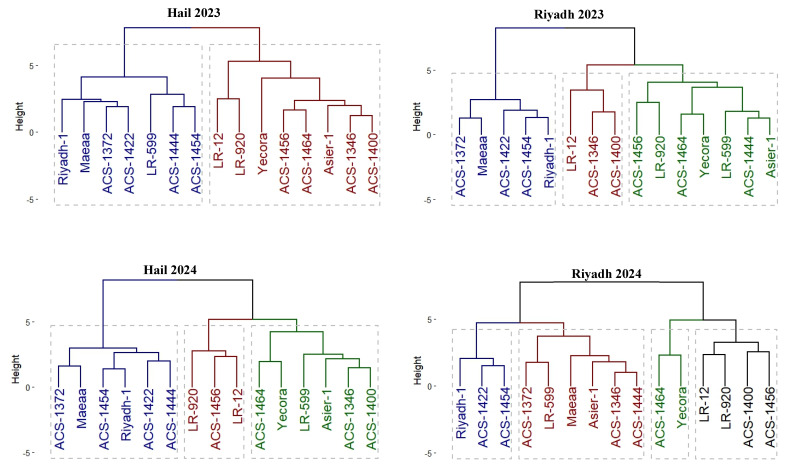
Hierarchical cluster analysis of fifteen bread wheat genotypes based on morphological, yield-contributing traits, and grain and biological yields under four environments: Hail-2023, Riyadh-2023, Hail-2024, and Riyadh-2024.

Hierarchical cluster analysis classified the evaluated wheat genotypes into distinct groups according to their agronomic performance in each environment (Figure 2). In Hail 2023, the first cluster comprised Riyadh-1, Maeaa, ACS-1372, ACS-1422, LR-599, ACS-1444, and ACS-1454. This group was characterized by superior agronomic performance. In contrast, the second cluster consists of LR-12, LR-920, Yecora, ACS-1456, ACS-1464, Asier-1, ACS-1346, and ACS-1400. This group showed lower productivity. LR-12 and LR-920 exhibited reduced grain yield despite their longer growth duration and taller plant stature. In Riyadh 2023, the first cluster included ACS-1372, Maeaa, ACS-1422, ACS-1454, and Riyadh-1. These genotypes represented the highest-performing group under the warmer environment. A second intermediate cluster consisted of LR-12, ACS-1346, and ACS-1400. The third cluster, including ACS-1456, LR-920, ACS-1464, Yecora, LR-599, ACS-1444, and Asier-1, exhibited lower agronomic performance. Under Hail 2024, the first cluster comprised ACS-1372, Maeaa, ACS-1454, Riyadh-1, ACS-1422, and ACS-1444. These genotypes represented the most productive group. These genotypes consistently produced high grain and biological yields, along with favorable values for spike density and grain weight. The second cluster included LR-920, ACS-1456, and LR-12. These genotypes displayed intermediate performance. The third cluster comprised ACS-1464, Yecora, LR-599, Asier-1, ACS-1346, and ACS-1400. They recorded lower agronomic performance. In Riyadh 2024, Riyadh-1, ACS-1422, and ACS-1454 formed a high-performing cluster. These genotypes combine superior grain and biological yields as well as spike density and grain weight. The second cluster included ACS-1372, LR-599, Maeaa, Asier-1, ACS-1346, and ACS-1444. These genotypes exhibited intermediate to high agronomic performance. The third cluster consisted of ACS-1464 and Yecora. They were characterized by moderate productivity. The fourth cluster included LR-12, LR-920, ACS-1400, and ACS-1456. They represented the least productive genotypes.

### 2.4. Principal Component Analysis Across Environments

**Figure 3:**
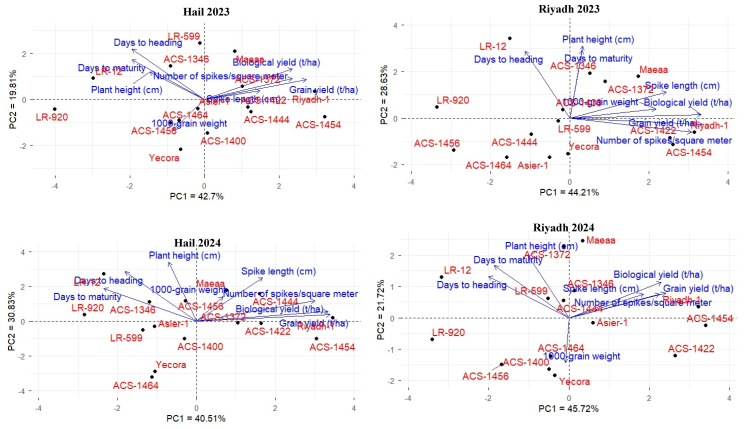
Principal component analysis biplots showing the associations among phenological, morphological, yield-contributing traits, grain, and biological yields of 15 bread wheat genotypes across four environments: Hail-2023, Riyadh-2023, Hail-2024, and Riyadh-2024.

Principal component analysis (PCA) was applied to examine the multivariate relationships among the evaluated wheat genotypes and the agronomic traits studied (Figure 3). In Hail 2023, the first two PCs explained 62.51% of the total variation (42.70% by PC1 and 19.81% by PC2). Grain yield, biological yield, spike length, and number of spikes/m^2^ were strongly and positively associated with PC1. Riyadh-1, ACS-1422, ACS-1372, ACS-1444, ACS-1454, and Maeaa were positioned in the direction of these vectors. This trend indicated their favorable yield-related characteristics. In contrast, LR-920 and LR-12 were located on the opposite negative side. This trend reflected their relatively lower productivity. Furthermore, LR-12, LR-599, and ACS-1346 were closely associated with days to heading and days to maturity. ACS-1456 and ACS-1464 showed a stronger association with 1000-grain weight. In Riyadh 2023, the first two PCs explained 72.84% of the total variation (44.21% by PC1 and 28.63% by PC2). Riyadh-1, ACS-1454, ACS-1422, ACS-1372, and Maeaa were located near the vectors of yield traits. This trend confirmed their superior agronomic performance under the warmer environmental conditions of Riyadh. Conversely, LR-12, LR-920, ACS-1456, ACS-1444, ACS-1464, and Asier-1 were positioned away from the yield-related vectors on the opposite negative side. In addition, LR-12 and ACS-1346 were more closely associated with days to heading and days to maturity. Under Hail 2024, the first two PCs explained 71.34% of the total variation (40.51% by PC1 and 30.83% by PC2). Riyadh-1, ACS-1444, ACS-1422, ACS-1454, and ACS-1372 were positioned near the vector of yield traits. In contrast, LR-920, LR-12, LR-599, ACS-1346, ACS-1464, and Yecora were located on the opposite negative side. LR-12, LR-920, and ACS-1346 were closely associated with days to heading and days to maturity. In Riyadh 2024, the first two PCs explained 67.44% of the total variation (45.72% by PC1 and 21.72% by PC2). Riyadh-1, ACS-1454, and ACS-1422 were positioned closest to the yield-trait vectors under warmer environmental conditions. Conversely, LR-920 and LR-12 were located on the opposite negative side and exhibited weaker associations with the yield-related traits. In addition, ACS-1464, ACS-1400, and Yecora showed a closer association with 1000-grain weight. Across environments, grain yield consistently exhibited strong positive associations with biological yield, number of spikes/m^2^ and spike length. This indicated that these traits were the primary determinants of productivity. Days to heading and days to maturity were generally separated from the yield-related traits.

### 2.5. Heatmap Analysis of Genotype Performance Across Environments

**Figure 4:**
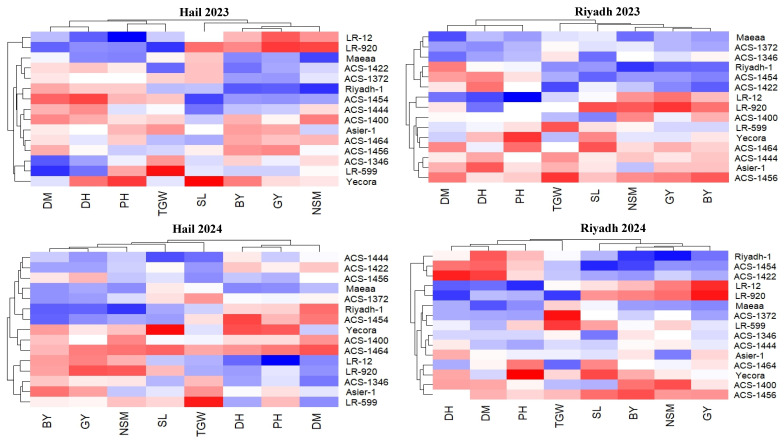
Hierarchical heatmap clustering of fifteen bread wheat genotypes based on phenological, morphological, and yield-contributing traits, as well as grain and biological yields, across four environments: Hail-2023, Riyadh-2023, Hail-2024, and Riyadh-2024. DH, days to heading; DM, days to physiological maturity; PH, plant height; SL, spike length; NSM, number of spikes/m^2^; TGW, 1000-grain weight; GY, grain yield; and BY, biological yield. The color scale ranges from red, representing lower values, through white, representing intermediate values, to blue, representing higher values.

The heatmap analysis provided an overview of genotype performance across environments using all studied traits (Figure 4). In Hail 2023, the heatmap separated the genotypes into distinct performance groups. Maeaa, ACS-1422, ACS-1372, Riyadh-1, and ACS-1454 were characterized by high grain yield, biological yield, and number of spikes/m^2^ as indicated by blue coloration. They displayed relatively early phenology as indicated by red coloration. ACS-1454 and ACS-1444 exhibited high spike numbers/m^2^ and spike length, as well as early phenology. In contrast, LR-12 and LR-920 were characterized by high values of days to heading and days to maturity but low values of grain and biological yields. In addition, LR-12 and LR-920, ACS-1422, Riyadh-1, ACS-1464, ACS-1456, and Yecora displayed relatively high thousand-grain weight. Under Riyadh 2023, Riyadh-1, ACS-1454, ACS-1422, ACS-1372, and Maeaa displayed high agronomic performance. These genotypes demonstrated their adaptation to the warmer environment. In Hail 2024, Riyadh-1, ACS-1454, ACS-1372, Maeaa, ACS-1422, and ACS-1444 recorded high yield traits. In Riyadh 2024, Riyadh-1, ACS-1454, ACS-1422, Maeaa, and ACS-1372 exhibited high-yielding traits under warmer conditions. Conversely, LR-12, LR-920, ACS-1400, and ACS-1456 exhibited reduced values for several yield-contributing traits. The heatmap also demonstrated that grain yield was associated with high biological yield, number of spikes/m^2^ and spike length. Long phenological duration was associated with reduced productivity. These findings confirmed that spike production, biomass accumulation, and spike architecture were the principal determinants of grain yield under arid environments.

### 2.6. Yield Stability

**Figure 5:**
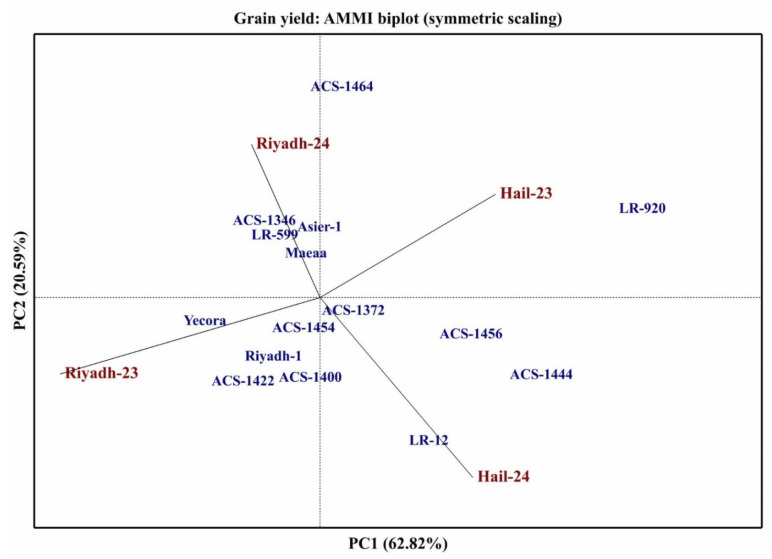
AMMI biplot of genotype-by-environment interaction for grain yield of 15 bread wheat genotypes across four environments: Riyadh 2023, Riyadh 2024, Hail 2023, and Hail 2024.

The additive main effects and multiplicative interaction (AMMI) biplot for grain yield explained 62.82% of the interaction variation by PC1 and 20.55% by PC2 (Figure 5). The distribution of genotypes on the biplot indicated differences in grain yield stability across the tested environments. Genotypes located near the origin exhibited smaller interaction effects across environments. Among these, ACS-1372, ACS-1454, Riyadh-1, ACS-1400, ACS-1422, and Maeaa were positioned close to the biplot origin. Conversely, genotypes positioned farther from the origin exhibited stronger interaction effects. ACS-1464, LR-920, LR-12, and ACS-1444 showed the largest interaction scores. They contributed strongly to the genotype-by-environment interaction. The four environments were clearly differentiated on the biplot. Hail-23 and Hail-24 were located on the positive side of PC1. Riyadh-23 and Riyadh-24 were positioned on the negative side of PC1. Specific adaptation patterns were evident from the proximity between genotypes and environments. This proximity indicated a favorable adaptation to this environment. LR-920 was closely associated with Hail-23, and LR-12 was associated with Hail-24. ACS-1464 was more closely related to Riyadh-24. On the other hand, Yecora was located nearer to Riyadh-23.

### 2.7. Grain Quality

Grain quality traits were evaluated using grain samples collected from the Riyadh experimental site during the 2023/2024 growing season. Therefore, the results presented below describe genotypic variation under a single production environment. Considerable variation was observed among the evaluated wheat genotypes for grain quality traits (Table 4). Protein, wet gluten, dry gluten, gluten index, fiber, and fat contents were assessed. Protein content ranged from 13.49% to 19.67%. The local landraces generally exhibited superior protein concentration. LR-12 and LR-599 recorded the highest protein content (19.67 and 18.92%), followed by Yecora (18.14%). In contrast, Riyadh-1, ACS-1454, and ACS-1422 recorded lower protein contents (13.49–14.54). Wet gluten ranged from 32.21% to 48.00%. Dry gluten varied from 10.38% to 15.80%. LR-12 and LR-599 exhibited the highest gluten contents. The gluten index ranged from 61.76% to 95.90%. Riyadh-1 possessed the highest gluten index (95.90%). It was followed by Yecora (95.10%), ACS-1456 (93.00%), and ACS-1346 (91.98%). These results indicated superior gluten strength and quality in these genotypes. In contrast, LR-920 and ACS-1422 recorded the lowest gluten index values (61.76% and 63.60%, respectively). This suggests weaker gluten properties despite their moderate protein levels. Fiber content ranged from 2.05% to 3.41%. Total fat content varied from 1.34% to 2.77%. Asier-1, LR-12, and ACS-1346 exhibited relatively high fiber contents. ACS-1400, Asier-1, and LR-12 recorded the highest total fat contents.

## 3. Discussion

The present study demonstrated that environmental conditions strongly influenced the phenological, morphological, and yield traits of bread wheat. The greater contribution of location compared with year effects suggests that spatial environmental variation between Hail and Riyadh was more influential than seasonal fluctuations. This can be attributed to the differences in temperature regimes between the two locations. These differences created contrasting conditions for crop growth and development. Riyadh showed reduced performance for all agronomic traits. Higher temperatures in Riyadh accelerated crop development and shortened grain-filling duration. These results emphasize the critical role of temperature in wheat productivity under arid conditions. Likewise, Lekhana et al. [8], Ullah et al. [24], and Li et al. [25] reported that elevated temperatures during reproductive development accelerate senescence. In addition, elevated temperatures reduce photosynthetic activity, impair spike fertility, shorten grain-filling duration, and ultimately decrease grain weight and yield. The evaluated germplasm exhibited significant differences for days to heading and maturity. The local landraces displayed later heading and maturity than the advanced lines and released cultivars. In contrast, ACS-1454, ACS-1422, ACS-1400, ACS-1464, Asier-1, and Riyadh-1 represented desirable adaptation mechanisms for arid and heat-prone environments. The superior performance of the early-maturing ACSAD lines suggests that phenological escape was an important adaptation mechanism under the warmer Riyadh environment. Earlier heading and maturity shortened the exposure of flowering and grain filling to high temperatures. Consequently, this reduces heat-induced reproductive damage and allows for more efficient assimilate partitioning during grain filling. Such early phenology is particularly advantageous in arid environments where terminal heat stress frequently limits wheat productivity. Similarly, Gulino and Lopes [18] and Collins and Chenu [26] demonstrated that earliness is an effective adaptive strategy under increasing temperatures. The local landraces LR-12 and LR-920 produced taller plant stature across environments, whereas ACSAD lines exhibited semi-dwarf growth habits. Reduced plant height is associated with improved lodging resistance and increased grain production [27]. In addition, ACS-1454, ACS-1444, and ACS-1346 exhibited superior spike length. This trait is often associated with increased sink capacity. A similar association between spike architecture and grain yield was reported in wheat by Li et al. [28] and Abbai et al. [29].

The results revealed considerable variation among genotypes for spike density and grain weight. Riyadh-1, Maeaa, and ACS-1454 produced higher numbers of spikes per square meter. The consistently high number of productive spikes of these genotypes appears to be primarily associated with their ability to maintain high grain yields across contrasting environments. Under arid conditions, spike density represents a major determinant of sink capacity and contributes to yield stability. These findings indicate that maintaining productive tillers under environmental stress is an important breeding target for improving wheat adaptation. Similar relationships between spike density and grain yield were reported under stress-prone environments by Boulacel et al. [30] and Groli et al. [31]. ACS-1422 and LR-920 exhibited high thousand-grain weight across environments. Grain weight plays a crucial role under environmental constraints [32]. ACS-1422 combined high grain weight with favorable spike density. These findings indicate the importance of balancing sink establishment and grain-filling efficiency in arid and heat-prone environments. Riyadh-1, ACS-1454, ACS-1422, ACS-1372, and Maeaa achieved superior grain yields across environments. They combined adaptive phenology, high spike density, grain weight, and biomass production. The greater grain yield under the Hail environment likely resulted from the extended vegetative and reproductive growth periods. This promoted canopy development, radiation interception, and assimilate production. This additional biomass supported longer grain filling and contributed to greater grain weight and final grain yield. Conversely, the warmer conditions in Riyadh shortened crop duration and reduced biomass production. This emphasizes the importance of selecting genotypes that maintain efficient biomass partitioning under terminal heat stress. Similar findings were reported by Menzir et al. [33] and Muhammad et al. [34] in wheat breeding. They elucidated that high yield is associated with optimization of multiple yield-contributing traits. In contrast, the local landraces produced lower grain yields despite their longer growth duration and taller plant stature, suggesting that prolonged vegetative growth alone is insufficient to improve grain productivity [35].

The significant genotype-by-environment interactions indicate that genotype performance varied across environments. The genotype-by-location interaction was greater than the genotype-by-year interaction. This greater magnitude suggests that spatial environmental differences contributed more to phenotypic variation than seasonal fluctuations. Similar findings have been reported in multi-environment trials by Sousa et al. [36] and Mansour et al. [37]. These results emphasize the importance of multi-location testing for assessing genotype adaptation. Furthermore, the genotype-by-location-by-year interaction was significant, indicating that genotype performance cannot be predicted from a single location or growing season. Therefore, evaluating breeding materials across diverse locations and multiple years remains essential, allows for the identification of high-yielding and stable genotypes.

The multivariate analyses provided insights into the relationships among genotypes and traits under arid environments [38,39,40]. Cluster analysis grouped the evaluated germplasm into distinct clusters. The cluster separated high-yielding genotypes from the local landraces. These results reflected differences among evaluated genotypes in phenology, plant architecture, and yield traits. Identifying genetically divergent groups is valuable for parent selection. Similar clustering patterns were reported in wheat germplasm collections by Corrêa et al. [41] and Gharib et al. [42]. They disclosed that the cluster analysis effectively discriminated between adapted breeding materials. The principal component biplot further revealed the relationships among genotypes and the studied traits. Genotypes located near vectors of yield traits were characterized by high productivity. Riyadh-1, ACS-1454, ACS-1422, ACS-1372, and Maeaa were among the most productive genotypes. LR-12, LR-920, and LR-599 are associated with longer growth duration and greater plant height. Grain yield, biological yield, spike density, and grain weight were strongly associated, confirming the importance of these traits as major contributors in arid environments. Similar relationships among yield components were deduced by Jaenisch et al. [43] and Kondić et al. [44]. They identified these traits as key determinants of grain yield across contrasting environments. The heatmap analysis complemented the cluster and PC results. It provided a comprehensive visualization of genotype–trait relationships. The heatmap distinguished superior genotypes characterized by high values for yield traits. Furthermore, the heatmap clustering agreed with the dendrogram and PC grouping, confirming the robustness of the observed patterns. The results of multivariate approaches demonstrate their effectiveness for identifying superior genotypes. These tools are useful for explaining trait associations in breeding datasets. Similarly, Mulugeta et al. [45] and Mulugeta et al. [46] demonstrated the value of multivariate analyses for classifying wheat germplasm and supporting selection decisions.

Yield stability is a critical criterion for cultivar selection under arid environments. The stability analysis revealed considerable differences among the tested genotypes across environments. Riyadh-1, ACS-1454, ACS-1422, ACS-1372, and Maeaa exhibited relatively stable responses across locations and seasons, indicating their broad adaptation to the target environments. The stability of these genotypes suggests the presence of favorable genetic mechanisms under contrasting environmental conditions. These genotypes combined high-yield performance with yield stability. Similar results have been reported by Abdi et al. [47] and Adhikari et al. [48]. They disclosed that genotypes with high yield and stability provide great value for future breeding programs.

The evaluated germplasm exhibited substantial variation for grain quality traits. These variations reflect the diverse genetic backgrounds of the studied materials. The local landraces LR-12 and LR-599 exhibited higher protein, wet gluten, and dry gluten contents than improved genotypes, indicating their potential as valuable sources of superior bread-making quality. In contrast, Riyadh-1, Yecora, and ACSAD lines exhibited relatively high gluten index values, indicating stronger gluten characteristics. These properties reveal superior dough strength despite their lower protein concentrations. Accordingly, grain quality is not determined only by protein quantity but also by protein composition and gluten structure. Similar relationships were reported by Zadeike et al. [49] and Zhao et al. [50]. They showed that gluten functionality is influenced by the balance between glutenin and gliadin proteins rather than by total protein concentration alone. The observed differences between grain productivity and grain-quality traits illustrate an important breeding consideration. While recently released cultivars and advanced breeding lines exhibited superior grain yield and adaptation, the Saudi landraces possessed higher protein and gluten contents. This complementarity reflects the commonly reported trade-off between grain yield and protein concentration. Increased starch accumulation in high-yielding genotypes may dilute grain protein content. Consequently, the local landraces represent valuable parental resources for improving end-use quality, while elite breeding materials provide superior adaptation and productivity.

This study provides new insights into wheat improvement under arid conditions by integrating recently released Saudi cultivars, indigenous Saudi landraces, advanced ACSAD breeding lines, and the historically important CIMMYT-derived cultivar Yecora within a single multi-environment framework. This unique germplasm combination enabled a direct comparison of traditional genetic resources, elite breeding materials, and commercial cultivars. This combination revealed sources of early maturity, adaptation, productivity, stability, and grain quality. The contrasting environments of Riyadh and Hail allowed for the identification of genotype-specific responses to major climatic constraints typical of arid wheat production systems. This thereby improve understanding of adaptation mechanisms under heat stress and arid conditions. The superior performance of Riyadh-1 together with ACS-1454, ACS-1422, and ACS-1372 demonstrates the effectiveness of breeding efforts in developing cultivars adapted to Saudi environments. The favorable grain-quality characteristics and adaptive traits observed in the local landraces highlight their continuing value as parental resources for broadening the genetic base of future breeding populations. Therefore, this study provides an evidence-based framework for cultivar recommendation, parental selection, and the strategic use of indigenous germplasm in the Saudi national wheat breeding program. Consequently, it contributes to the development of climate-resilient, high-yielding wheat cultivars for arid environments.

## 4. Materials and Methods

### 4.1. Plant Materials

Fifteen bread wheat (*Triticum aestivum* L.) genotypes were evaluated in this study. The germplasm set consisted of eight advanced breeding lines developed by the Arab Center for the Studies of Arid Zones and Dry Lands (ACSAD), three Saudi local landraces (LR-12, LR-599, and LR-920), three recently released cultivars (Asier-1, Riyadh-1, and Maeaa), and the CIMMYT cultivar Yecora. The selected materials represent a broad range of genetic diversity. The ACSAD advanced lines were included as promising breeding materials. The local landraces are valuable genetic resources due to their adaptation to local growing conditions and their potential to contribute useful alleles for stress adaptation and resilience. The recently released cultivars were included as commercial varieties for comparison. Yecora was used as a recognized cultivar with proven adaptation to dry environments. Detailed information on the origin, pedigree, and classification of the evaluated genotypes is presented in Table S4.

### 4.2. Experimental Locations

Field experiments were conducted at two contrasting environments in Saudi Arabia: Riyadh (24°38′ N, 46°43′ E) and Hail (27°31′00″ N, 41°41′00″ E). Both locations are characterized by arid climates, however, there are differences in temperature, relative humidity, and precipitation. Riyadh had higher temperatures than Hail throughout the growing seasons. Maximum temperatures in Riyadh ranged from 17.8 to 44.7 °C. Minimum temperatures varied from 5.4 to 25.5 °C, whereas Hail exhibited relatively cooler conditions, with maximum temperatures ranging from 15.5 to 39.4 °C and minimum temperatures from 1.4 to 19.8 °C. Relative humidity was generally high during the winter months at both locations, but was higher in Hail than in Riyadh. Riyadh received lower rainfall. These climatic differences create contrasting agroecological conditions to evaluate the tested genotypes. Meteorological data of the two locations throughout the growing seasons are presented in Table S5. Soil samples were collected from both locations and analyzed. The soil at Riyadh was classified as sandy loam, comprising 79.1% sand, 12.5% silt, and 8.4% clay. The soil at Hail was loamy sand, comprising 88.2% sand, 5.0% silt, and 6.8% clay. Both soils were slightly alkaline (pH was 7.84 in Riyadh and 7.78 in Hail). Electrical conductivity (EC) was low at both sites. However, Riyadh exhibited a higher EC (0.75 dS m^−1^) than Hail (0.25 dS m^−1^). Riyadh soil contained a higher concentration of calcium carbonate (37.5%) than Hail soil (8.5%). Nutrient availability differed between locations. Riyadh showed higher total nitrogen (38 ppm) and available phosphorus (108.9 ppm) than Hail (5 and 14.5 ppm, respectively). Exchangeable potassium and sodium levels were slightly higher in Hail (122 and 84 ppm, respectively) than in Riyadh (110 and 70 ppm, respectively).

### 4.3. Experimental Design and Crop Management

Field experiments were conducted at each location using a Randomized Complete Block Design with three replications. Each experimental plot consisted of six rows, each 5 m in length, with a row spacing of 20 cm. The evaluated genotypes were sown during the first week of December in both growing seasons at the two experimental locations. This sowing date corresponds to the recommended planting date for bread wheat in Saudi Arabia. It allowed the crop to experience the natural temperature gradients prevailing at the two locations throughout vegetative and reproductive development. Seeds were sown at 400 seeds/m^2^ to ensure adequate plant establishment. Phosphorus was supplied at 65 kg P_2_O_5_/ha, nitrogen at 200 kg N/ha, and potassium at 80 kg K_2_O/ha. Sprinkler irrigation was applied throughout the growing season at both experimental sites to avoid water limitation. Therefore, differences among environments were primarily attributable to climatic variation, particularly temperature, rather than to drought stress. The harvesting was carried out in mid-April at Riyadh and in early May at Hail, corresponding to the physiological maturity of the evaluated genotypes.

### 4.4. Traits Measured

Days to heading was recorded as the number of days from sowing to the stage when approximately 50% of the spikes had fully emerged. Days to physiological maturity was determined as the number of days from sowing until approximately 90% of the spikes had reached physiological maturity. It was determined by the loss of green color in the peduncle and spike. Plant height (cm) was measured at maturity from the soil surface to the tip of the spike, excluding awns. Spike length (cm) was measured on ten randomly selected spikes collected from each plot at maturity. The number of spikes per square meter was determined by counting all productive spikes. Thousand-grain weight (g) was measured by weighing a random sample of 1000 kernels. Grain yield (t/ha) and biological yield (t/ha) were determined by harvesting the central four rows of each plot to minimize border effects. The harvested material was threshed, cleaned, and weighed separately to determine grain yield and total aboveground biomass. Grain yield and biological yield were converted to tons per hectare. Grain quality analyses were performed using grain samples harvested from the Riyadh experimental site during the 2023/2024 growing season. This site was selected to provide a uniform production environment for comparative evaluation of genotype performance with respect to grain quality traits. Consequently, the grain quality data represent differences among genotypes under a single environment. Grain samples were collected separately from each replication after harvest. Each replication constituted one biological replicate. After cleaning and conditioning, grain samples were analyzed for protein content (dry basis), wet gluten, dry gluten, gluten index, ash, fiber, and total fat. Protein content was determined on a dry matter basis using standard cereal grain analysis procedures. The wet gluten, dry gluten, and gluten indices were measured using a gluten analyzer. Crude fiber content was determined using the standard acid–alkali digestion method. Total fat content was measured by solvent extraction. The reported values represent the mean ± standard deviation of three biological replicates. The genotype means were compared using Fisher’s LSD test (*p* ≤ 0.05) following a significant analysis of variance.

### 4.5. Statistical Analysis

The experiments at each environment were arranged in a randomized complete block design with three replications. A combined analysis of variance across environments was performed using GenStat 19th Edition (VSN International, Hemel Hempstead, UK). The statistical model was: Y_ijkl_ = μ + E_i_ + R(E)_j(i)_ + G_k_ + (GE)_ik+εijkl_, where Y_ijkl_ is the observed value, μ is the overall mean, E_i_ is the effect of the ith environment (location × year combination), R(E)_j(i)_ is the effect of the jth replication nested within the ith environment, G_k_ is the effect of the kth genotype, (GE)_ik_ is the genotype-by-environment interaction, and εijkl is the residual error. Genotype was considered a fixed effect because the objective was to compare a defined set of wheat genotypes, whereas environments and replications within environments were considered random effects to permit inference across representative target environments. The environmental effect was further partitioned into location (L), year (Y), and their interaction (L × Y). While the genotype × environment interaction was partitioned into G × L, G × Y, and G × L × Y. Mean separation was performed using Fisher’s Least Significant Difference (LSD) test at *p* ≤ 0.05. The LSD test was used to facilitate pairwise comparisons among genotype means within environments and among the main-effect means. Genotype adaptation and stability were assessed using AMMI, principal component analysis, hierarchical clustering, and heatmaps. Violin plots were generated in R software (version 4.6.1) using the ggplot2, dplyr, and rstatix packages. Hierarchical clustering was performed using the FactoMineR, factoextra, and ggplot2 packages. Principal component analysis was performed using the FactoMineR, factoextra, ggplot2, and ggrepel packages. Heatmap analysis was conducted using the gplots package. The Additive Main Effects and Multiplicative Interaction (AMMI) model was applied using Genstat version 19 [51].

## 5. Conclusions

This study demonstrated substantial genetic variation among fifteen bread wheat genotypes evaluated across two contrasting arid environments in Saudi Arabia. Environmental conditions, particularly temperature differences between Hail and Riyadh, significantly influenced phenological development, plant growth, yield components, and productivity. Significant genotype-by-environment interactions for the studied traits confirmed the differential responses of genotypes across environments. The ACSAD advanced lines exhibited superior earliness and favorable agronomic characteristics, whereas the local landraces maintained longer growth duration and taller plant stature. Riyadh-1 consistently exhibited superior grain and biological yields across environments. ACS-1454, ACS-1422, ACS-1372, and Maeaa also exhibited excellent productivity and adaptation across environments. These genotypes combined desirable phenological patterns and favorable yield components. These genotypes displayed stable performance under contrasting environmental conditions. Substantial variation was observed for grain use quality. In addition, the local landraces LR-12 and LR-599 represented valuable sources of adaptive diversity and superior protein and gluten contents, whereas Riyadh-1 and Yecora displayed stronger gluten quality, as reflected by their high gluten index values. These findings provide practical guidance for genotype selection and highlight the importance of integrating locally adapted genetic resources with advanced breeding materials to develop climate-resilient wheat cultivars for sustainable production in arid environments.

## Figures and Tables

**Table 1:** Mean squares from the analysis of variance (ANOVA) for the studied traits of fifteen wheat genotypes grown in four environments: Riyadh and Hail during the 2022–2023 and 2023–2024 growing seasons.

Source of Variation	df	Grain Yield (t/ha)	Biological Yield (t/ha)	No Spikes Per Square Meter	Thousand Grain Weight (g)
**Genotype (G)**	**14**	6.459 **	46.50 **	21,531 **	22.64 **
**Environment (E)**	**3**	21.75 **	207.4 **	129,789 **	583.2 **
**Replication/E**	**8**	**0.043**	**0.326**	**28.00**	**2.069**
*Location (L)*	*1*	58.73 **	528.7 **	350,612 **	1471 **
*Year (Y)*	*1*	5.961 **	83.53 **	35,546 **	261.7 **
*L × Y*	*1*	0.567 **	10.09 **	3209 **	16.27 *
**G × E**	**42**	0.161 **	1.691 **	885.4 **	5.186 **
*G × L*	*14*	0.241 **	2.901 **	1678 **	6.347 **
*G × Y*	*14*	0.129 *	1.458 **	764.9 **	4.784 **
*G × L × Y*	*14*	0.113 *	0.716 **	213.7 *	4.427 **
*PC1*	*16*	0.285 **	2.710 **	1690 **	6.520 **
*PC2*	*14*	0.201 **	1.370 **	585.0 **	6.760 **
**Residual**	**112**	**0.064**	**0.254**	**107.0**	**1.688**
**Total**	**179**	**0.95**	**7.684**	**4135**	**13.91**
Source of variation	df	Plant Height (cm)	Spike Length (cm)	Days to Heading	Days to Maturity
**Genotype (G)**	**14**	769.9 **	5.929 **	113.2 **	90.21 **
**Environment (E)**	**3**	1550 **	38.56 **	1043 **	1081 **
**Replication/E**	**8**	**7.508**	**0.402**	**7.436**	**1.842**
*Location(L)*	*1*	4091 **	108.7 **	2917 **	3060 **
*Year (Y)*	*1*	557.8 **	5.233 *	182.3 **	167.1 **
*L × Y*	*1*	*0.843 ns*	*1.798 ns*	29.34 *	17.05 *
**G × E**	**42**	30.01 **	0.643 *	8.730 **	9.370 **
*G × L*	*14*	50.59 **	1.123 **	17.07 **	13.89 **
*G × Y*	*14*	16.72 **	*0.359 ns*	*4.549 ns*	7.468 **
*G × L × Y*	*14*	22.70 **	*0.445 ns*	*4.570 ns*	6.754 **
*PC1*	*16*	47.10 **	1.036 **	15.70 **	12.20 **
*PC2*	*14*	23.70 **	*0.498 ns*	4.600 **	7.700 **
**Residual**	**112**	**6.273**	**0.582**	**2.745**	**2.691**
**Total**	**179**	**97.50**	**1.643**	**30.43**	**29.14**

Main sources of variation are shown in bold type. Values representing the decomposition of each main source of variation are shown in italics and separated by dashed lines. PC: Interaction explained by the principal components. ns: not significant, * *p* < 0.05; ** *p* < 0.01.

**Table 2:** Mean performance of fifteen bread wheat genotypes for agronomic and yield-related traits under Hail and Riyadh environments, averaged across the 2022/2023 and 2023/2024 growing seasons.

Genotype	Grain Yield (t/ha)		Biological Yield (t/ha)		No Spikes Per Square Meter		Thousand Grain Weight (g)	
	Hail	Riyadh	Hail	Riyadh	Hail	Riyadh	Hail	Riyadh
ACS-1346	**7.033 ± 0.50**	**6.165 ± 0.23**	18.85 ± 1.21	16.12 ± 0.75	489.7 ± 19.21	**415.7 ± 13.49**	43.81 ± 1.91	39.02 ± 1.52
ACS-1372	**7.690 ± 0.32**	**6.516 ± 0.18**	**21.93 ± 1.12**	**17.87 ± 1.16**	**514.7 ± 19.71**	**419.3 ± 20.64**	43.85 ± 2.6	37.10 ± 3.60
ACS-1400	6.996 ± 0.28	5.873 ± 0.38	18.53 ± 0.91	14.45 ± 0.84	455.5 ± 18.02	349.8 ± 25.58	44.87 ± 1.92	**40.59 ± 1.55**
ACS-1422	**7.641 ± 0.32**	**6.697 ± 0.44**	**22.15 ± 1.55**	**19.06 ± 1.49**	**520.0 ± 22.28**	**438.2 ± 20.18**	**46.94 ± 2.18**	**41.44 ± 2.22**
ACS-1444	**7.538 ± 0.36**	5.853 ± 0.22	**21.03 ± 1.20**	15.78 ± 0.45	**505.0 ± 11.50**	396.1 ± 8.64	**46.37 ± 1.33**	38.80 ± 0.89
ACS-1454	**7.761 ± 0.50**	**6.719 ± 0.37**	**22.41 ± 0.68**	**19.42 ± 0.57**	**537.9 ± 23.86**	**462.6 ± 19.74**	44.41 ± 1.81	**40.67 ± 1.24**
ACS-1456	6.425 ± 0.27	4.981 ± 0.19	18.54 ± 0.81	13.19 ± 0.61	**515.2 ± 11.95**	364.8 ± 9.60	**46.33 ± 1.40**	36.97 ± 0.62
ACS-1464	6.410 ± 0.53	5.455 ± 0.17	18.31 ± 0.87	15.96 ± 0.25	462.6 ± 24.27	407.9 ± 15.79	44.78 ± 3.43	**40.76 ± 0.81**
Asier-1	6.460 ± 0.39	5.463 ± 0.23	17.60 ± 1.50	15.51 ± 0.49	**522.4 ± 19.79**	**455.8 ± 7.30**	43.42 ± 2.26	38.84 ± 1.09
LR-12	6.111 ± 0.20	4.598 ± 0.32	18.24 ± 1.32	15.11 ± 0.49	466.7 ± 14.53	360.4 ± 12.79	**46.11 ± 1.68**	39.66 ± 2.05
LR-599	**7.160 ± 0.56**	**6.182 ± 0.29**	**20.19 ± 1.80**	**17.03 ± 1.18**	489.5 ± 21.84	401.6 ± 20.02	41.60 ± 2.24	36.10 ± 1.86
LR-920	5.869 ± 0.35	4.255 ± 0.12	17.82 ± 0.91	13.92 ± 0.17	435.8 ± 14.94	343.5 ± 13.34	**47.07 ± 2.64**	**41.23 ± 1.66**
Maeaa	**7.620 ± 0.31**	**6.621 ± 0.27**	**22.12 ± 1.40**	**18.39 ± 0.92**	**560.4 ± 26.86**	**471.6 ± 29.12**	**45.03 ± 1.95**	39.19 ± 2.13
Riyadh-1	**8.06 ± 0.36**	**7.031 ± 0.47**	**23.09 ± 1.19**	**19.79 ± 0.58**	**579.3 ± 24.26**	**511.6 ± 18.60**	**45.93 ± 2.08**	**40.40 ± 2.42**
Yecora	6.821 ± 0.25	**6.050 ± 0.29**	17.64 ± 0.72	15.43 ± 0.96	485.5 ± 29.64	**417.4 ± 25.04**	**45.54 ± 1.83**	39.55 ± 2.37
LSD	0.287		0.581		11.532		1.497	
Genotype	Plant Height		Spike Length		Days to Heading		Days to Maturity	
	(cm)		(cm)					
	Hail	Riyadh	Hail	Riyadh	Hail	Riyadh	Hail	Riyadh
ACS-1346	105.4 ± 3.20	97.60 ± 3.95	**13.62 ± 0.64**	**12.73 ± 0.63**	91.59 ± 1.41	82.78 ± 2.47	136.5 ± 1.80	126.6 ± 2.95
ACS-1372	103.7 ± 2.19	99.16 ± 2.43	12.84 ± 0.41	11.80 ± 0.51	**88.73 ± 2.07**	83.75 ± 1.72	**131.6 ± 1.79**	126.6 ± 1.52
ACS-1400	**100.1 ± 3.21**	**93.34 ± 2.79**	**13.44 ± 0.80**	**12.43 ± 0.60**	**86.86 ± 2.15**	**78.95 ± 2.56**	**128.8 ± 2.40**	**122.6 ± 2.36**
ACS-1422	**101.1 ± 3.50**	**92.28 ± 4.31**	12.94 ± 0.99	**12.04 ± 0.73**	**86.88 ± 2.28**	**74.78 ± 2.66**	**130.7 ± 2.61**	**120.8 ± 3.49**
ACS-1444	107.0 ± 2.79	95.79 ± 1.69	**14.37 ± 1.02**	11.73 ± 0.41	**86.70 ± 3.13**	**78.87 ± 1.48**	**131.3 ± 1.53**	124.5 ± 1.50
ACS-1454	96.60 ± 2.71	**90.55 ± 2.66**	**14.25 ± 1.03**	**13.06 ± 0.65**	**82.89 ± 2.98**	**76.42 ± 2.06**	**127.9 ± 2.49**	**120.2 ± 2.06**
ACS-1456	108.6 ± 2.34	**90.03 ± 1.53**	**13.25 ± 0.61**	10.85 ± 0.96	89.58 ± 1.80	**80.1 ± 1.03**	**131.1 ± 1.44**	**120.8 ± 1.78**
ACS-1464	**94.99 ± 6.20**	**83.78 ± 1.98**	12.92 ± 1.41	10.51 ± 0.50	**87.20 ± 4.24**	82.25 ± 1.07	**128.7 ± 4.35**	**122.0 ± 1.55**
Asier-1	**98.44 ± 4.42**	**92.49 ± 1.64**	**13.30 ± 0.73**	11.65 ± 1.11	**88.78 ± 1.65**	**76.67 ± 1.88**	132.3 ± 1.67	**122.7 ± 1.82**
LR-12	125.2 ± 2.92	110.2 ± 5.17	**13.44 ± 0.52**	11.55 ± 0.66	93.47 ± 1.65	86.12 ± 1.44	135.1 ± 1.17	128.0 ± 1.39
LR-599	**96.04 ± 1.43**	**90.8 ± 2.33**	**13.14 ± 1.03**	11.18 ± 0.47	92.13 ± 2.17	81.13 ± 1.54	136.7 ± 2.21	125.4 ± 1.48
LR-920	109.6 ± 5.69	95.59 ± 2.61	12.43 ± 0.57	10.53 ± 0.77	91.93 ± 1.69	86.01 ± 0.95	135.9 ± 2.46	124.5 ± 1.24
Maeaa	113.3 ± 2.95	101.0 ± 3.78	12.90 ± 0.74	11.78 ± 0.42	92.36 ± 1.67	83.04 ± 1.46	133.5 ± 2.43	128.8 ± 1.30
Riyadh-1	**98.50 ± 3.62**	**92.17 ± 5.38**	**13.76 ± 0.68**	**12.33 ± 0.57**	**87.16 ± 2.20**	**80.34 ± 1.77**	**128.5 ± 3.32**	**119.7 ± 1.91**
Yecora	**87.15 ± 2.20**	**77.87 ± 1.68**	11.41 ± 0.70	10.50 ± 0.84	**83.72 ± 3.01**	**78.05 ± 1.68**	133.5 ± 1.47	125.2 ± 1.40
LSD	2.882		0.863		2.002		1.856	

Values represent the means of the 2022/2023 and 2023/2024 growing seasons for each location. The favorable value for each trait in each location is shown in bold.

**Table 3:** Mean performance of fifteen bread wheat genotypes for grain and biological yields across two contrasting locations in Saudi Arabia (Riyadh and Hail) during the 2023 and 2024 growing seasons.

Genotype	Hail-23	Riyadh-23	Hail-24	Riyadh-24	Mean
	Grain Yield (t/ha)				
ACS-1346	7.401 ± 0.16	6.307 ± 0.17	6.663 ± 0.43	6.020 ± 0.19	6.598 ± 0.58
ACS-1372	7.943 ± 0.21	6.660 ± 0.12	7.437 ± 0.18	6.370 ± 0.16	7.103 ± 0.66
ACS-1400	7.210 ± 0.15	6.170 ± 0.15	6.780 ± 0.20	5.577 ± 0.28	6.434 ± 0.67
ACS-1422	7.863 ± 0.29	7.073 ± 0.16	7.420 ± 0.14	6.320 ± 0.17	7.169 ± 0.61
ACS-1444	7.607 ± 0.30	5.773 ± 0.29	7.467 ± 0.47	5.930 ± 0.14	6.694 ± 0.93
ACS-1454	7.983 ± 0.54	6.917 ± 0.32	7.537 ± 0.45	6.520 ± 0.34	7.239 ± 0.69
ACS-1456	6.510 ± 0.30	4.893 ± 0.24	6.340 ± 0.27	5.073 ± 0.12	5.704 ± 0.79
ACS-1464	6.867 ± 0.17	5.313 ± 0.18	5.953 ± 0.28	5.597 ± 0.11	5.933 ± 0.63
Asier-1	6.717 ± 0.38	5.443 ± 0.27	6.203 ± 0.21	5.487 ± 0.25	5.963 ± 0.60
LR-12	6.220 ± 0.20	4.807 ± 0.24	6.011 ± 0.16	4.387 ± 0.26	5.353 ± 0.83
LR-599	7.597 ± 0.33	6.413 ± 0.15	6.723 ± 0.32	5.953 ± 0.15	6.672 ± 0.66
LR-920	6.023 ± 0.12	4.243 ± 0.14	5.717 ± 0.47	4.267 ± 0.14	5.063 ± 0.88
Maeaa	7.883 ± 0.15	6.677 ± 0.37	7.360 ± 0.19	6.567 ± 0.18	7.123 ± 0.59
Riyadh-1	8.327 ± 0.29	7.367 ± 0.26	7.790 ± 0.14	6.693 ± 0.39	7.544 ± 0.67
Yecora	7.017 ± 0.12	6.287 ± 0.16	6.627 ± 0.16	5.810 ± 0.10	6.435 ± 0.48
Mean	7.278 ± 0.72	6.023 ± 0.91	6.801 ± 0.85	5.771 ± 0.74	
LSD_g0.05%_	0.305				
LSD_e0.05%_	0.221				
LSD_g×e0.05%_	0.608				
	Biological Yield (t/ha)				
ACS-1346	19.86 ± 0.41	16.65 ± 0.43	17.84 ± 0.65	15.59 ± 0.63	17.49 ± 1.72
ACS-1372	22.78 ± 0.92	18.87 ± 0.34	21.07 ± 0.29	16.86 ± 0.44	19.90 ± 2.38
ACS-1400	19.27 ± 0.50	15.19 ± 0.23	17.78 ± 0.40	13.70 ± 0.25	16.49 ± 2.29
ACS-1422	23.53 ± 0.20	20.41 ± 0.22	20.77 ± 0.49	17.71 ± 0.17	20.60 ± 2.17
ACS-1444	22.10 ± 0.23	15.67 ± 0.51	19.95 ± 0.28	15.89 ± 0.45	18.40 ± 2.87
ACS-1454	22.89 ± 0.55	19.72 ± 0.64	21.92 ± 0.36	19.11 ± 0.34	20.91 ± 1.67
ACS-1456	18.67 ± 0.88	13.24 ± 0.68	18.41 ± 0.91	13.14 ± 0.67	15.86 ± 2.88
ACS-1464	19.06 ± 0.35	15.81 ± 0.28	17.55 ± 0.20	16.10 ± 0.11	17.13 ± 1.37
Asier-1	18.90 ± 0.37	15.46 ± 0.53	16.30 ± 0.67	15.56 ± 0.55	16.55 ± 1.53
LR-12	19.40 ± 0.53	15.35 ± 0.53	17.08 ± 0.28	14.87 ± 0.36	16.67 ± 1.89
LR-599	21.78 ± 0.42	17.92 ± 0.79	18.60 ± 0.60	16.14 ± 0.70	18.62 ± 2.20
LR-920	18.42 ± 0.47	13.82 ± 0.17	17.22 ± 0.87	14.01 ± 0.13	15.87 ± 2.13
Maeaa	23.19 ± 0.81	19.09 ± 0.29	21.04 ± 0.90	17.68 ± 0.75	20.25 ± 2.25
Riyadh-1	24.13 ± 0.38	20.23 ± 0.32	22.05 ± 0.36	19.36 ± 0.43	21.44 ± 1.94
Yecora	18.21 ± 0.31	16.24 ± 0.34	17.07 ± 0.48	14.62 ± 0.45	16.53 ± 1.41
Mean	20.81 ± 2.13	16.91 ± 2.29	18.98 ± 1.97	16.02 ± 1.86	
LSD_g0.05%_	0.607				
LSD_e0.05%_	0.537				
LSD_g×e0.05%_	1.033				

Values are presented as mean ± standard deviation. LSD g, least significant difference for comparing genotype means at the 5% probability level; LSD e, least significant difference for comparing environment means at the 5% probability level; LSD g × e, least significant difference for comparing genotype-by-environment interaction means at the 5% probability level.

**Table 4:** Grain quality characteristics of the evaluated bread wheat genotypes, including protein content, gluten properties, ash, fiber, and total fat content.

Genotype	Protein Dry Basis (%)	Wet Gluten (%)	Dry Gluten (%)
ACS-1346	16.18 ± 0.57	39.02 ± 1.00	12.63 ± 0.24
ACS-1372	15.26 ± 0.44	32.52 ± 1.36	10.98 ± 0.40
ACS-1400	16.03 ± 0.60	40.48 ± 0.94	13.19 ± 0.48
ACS-1422	14.54 ± 0.67	32.66 ± 0.83	11.14 ± 0.39
ACS-1444	16.12 ± 0.43	41.34 ± 0.71	13.43 ± 0.46
ACS-1454	13.59 ± 0.34	35.38 ± 1.38	11.45 ± 0.35
ACS-1456	15.44 ± 0.41	41.71 ± 0.75	13.09 ± 0.65
ACS-1464	15.41 ± 0.69	33.99 ± 0.89	10.66 ± 0.60
Asier-1	16.25 ± 0.41	36.66 ± 1.38	12.05 ± 0.24
LR-12	19.67 ± 0.68	48.00 ± 1.08	15.80 ± 0.53
LR-599	18.92 ± 0.44	46.00 ± 0.74	15.20 ± 0.46
LR-920	15.86 ± 0.68	35.59 ± 1.13	11.60 ± 0.52
Maeaa	16.08 ± 0.36	36.35 ± 1.06	13.15 ± 0.63
Riyadh-1	13.49 ± 0.25	32.21 ± 0.88	10.38 ± 0.61
Yecora	18.14 ± 0.47	41.18 ± 1.23	13.24 ± 0.57
LSD_g0.05%_	0.863	1.814	0.849
Genotype	Gluten Index	Fiber (%)	Fat (%)
ACS-1346	91.98 ± 1.40	3.29 ± 0.22	1.34 ± 0.35
ACS-1372	76.75 ± 1.38	2.93 ± 0.19	1.90 ± 0.20
ACS-1400	79.08 ± 0.90	2.47 ± 0.52	2.77 ± 0.14
ACS-1422	63.60 ± 1.21	2.35 ± 0.36	1.61 ± 0.26
ACS-1444	85.27 ± 1.06	2.51 ± 0.42	1.67 ± 0.16
ACS-1454	74.10 ± 0.90	2.15 ± 0.48	1.95 ± 0.19
ACS-1456	93.00 ± 1.70	2.28 ± 0.49	1.42 ± 0.35
ACS-1464	87.33 ± 0.93	2.05 ± 0.41	1.65 ± 0.18
Asier-1	90.73 ± 1.23	3.41 ± 0.31	2.33 ± 0.14
LR-12	84.79 ± 1.01	3.31 ± 0.46	2.31 ± 0.32
LR-599	79.10 ± 1.17	2.79 ± 0.49	1.96 ± 0.13
LR-920	61.76 ± 0.88	2.35 ± 0.27	2.26 ± 0.21
Maeaa	82.45 ± 1.69	2.65 ± 0.46	1.85 ± 0.25
Riyadh-1	95.90 ± 1.64	2.82 ± 0.26	2.07 ± 0.30
Yecora	95.10 ± 1.06	2.42 ± 0.19	1.63 ± 0.28
LSD_g0.05%_	2.042	0.665	0.339

## Data Availability

The data presented in this study are available from the corresponding author upon request. The dataset was generated as part of an ongoing national wheat breeding program conducted by the Seed Center and Plant Genetic Resources Bank, Ministry of Environment, Water and Agriculture (MEWA), Saudi Arabia. Supporting data have been provided to the journal during the submission process.

## Supplementary Materials

The following supporting information can be downloaded at https://www.mdpi.com/article/10.3390/plants15152385/s1: Table S1: Mean performance of fifteen bread wheat genotypes for number of spikes per square meter and thousand grain weight across two contrasting locations in Saudi Arabia (Riyadh and Hail) during the 2023 and 2024 growing seasons; Table S2: Mean performance of fifteen bread wheat genotypes for plant height and spike length across two contrasting locations in Saudi Arabia (Riyadh and Hail) during the 2023 and 2024 growing seasons; Table S3: Mean performance of fifteen bread wheat genotypes for days to heading and days to physiological maturity across two contrasting locations in Saudi Arabia (Riyadh and Hail) during the 2023 and 2024 growing seasons; Table S4: Pedigree, classification, origin, and year of release of the fifteen wheat genotypes evaluated in the present study; Table S5: Monthly climatic data at Riyadh and Hail during the 2022–2023 and 2023–2024 growing seasons.

## Abbreviations

The following abbreviations are used in this manuscript:

ACSAD	Arab Center for the Studies of Arid Zones and Dry Lands
CIMMYT	International Maize and Wheat Improvement Center
MEWA	Ministry of Environment, Water and Agriculture
EC	Electrical Conductivity
RCBD	Randomized Complete Block Design
LSD	Least Significant Difference
DH	Days to Heading
DM	Days to Maturity
PH	Plant Height
SL	Spike Length
NSM	Number of Spikes per Square Meter
TGW	Thousand-Grain Weight
BY	Biological Yield
GY	Grain Yield
ANOVA	Analysis of Variance
E	Environment
G	Genotype
GEI	Genotype × Environment Interaction
L	L: Location
Y	Year
AMMI	Additive Main Effects and Multiplicative Interaction
PC	Principal Component
