# Supplementary material for: Climate-Resilient Bread Wheat for Arid Environments: Adaptation and Yield Performance of Diverse Genotypes Across Contrasting Growing Conditions
Source: Plants (Basel). 2026 Aug 3;15(15):2385. doi: 10.3390/plants15152385 (PMC13467808; doi:10.3390/plants15152385)
Supplement: Supplementary file 1 [file plants-15-02385-s001.zip › plants-4426346-supplementary.pdf]

**Table S1**. Mean performance of fifteen bread wheat genotypes for number of spikes per square meter and thousand grain weight across two contrasting locations in Saudi Arabia (Riyadh and Hail) during the 2023 and 2024 growing seasons.

| Genotype | Hail-23 | Riyadh-23 | Hail-24 | Riyadh-24 | Mean |
|---|---|---|---|---|---|
| | | **Number of spikes per square meter** | | | |
| ACS-1346 | 506.3±8.33 | 425.0±5.57 | 473.1±5.03 | 406.4±12.79 | **452.7±41.78** |
| ACS-1372 | 531.3±4.73 | 435.7±14.01 | 498.1±11.01 | 402.9±8.05 | **467.0±53.42** |
| ACS-1400 | 470.5±5.29 | 362.7±23.86 | 440.5±10.40 | 336.9±23.87 | **402.7±59.11** |
| ACS-1422 | 538.5±11.54 | 453.7±11.06 | 501.4±8.88 | 422.6±13.09 | **479.0±47.29** |
| ACS-1444 | 505.9±5.80 | 394.5±10.50 | 504.0±10.77 | 397.7±10.31 | **450.5±56.97** |
| ACS-1454 | 558.5±11.54 | 478.5±11.13 | 517.2±11.59 | 446.6±9.28 | **500.2±44.53** |
| ACS-1456 | 523.8±7.91 | 365.4±10.65 | 506.6±8.54 | 364.1±10.75 | **440.0±79.25** |
| ACS-1464 | 493.1±9.58 | 405.7±5.59 | 432.0±6.48 | 410.0±6.24 | **435.2±36.92** |
| Asier-1 | 539.5±3.47 | 455.8±6.19 | 505.3±9.61 | 455.8±7.83 | **489.1±37.37** |
| LR-12 | 477.5±9.57 | 366.8±12.56 | 455.8±9.05 | 353.9±11.27 | **413.5±57.03** |
| LR-599 | 517.7±10.33 | 417.7±9.50 | 461.3±6.39 | 385.4±11.41 | **445.5±52.48** |
| LR-920 | 448.1±9.00 | 338.7±14.76 | 423.5±4.92 | 348.3±12.58 | **389.7±50.03** |
| Maeaa | 593.5±7.84 | 497.5±11.18 | 527.3±6.58 | 445.7±7.70 | **516.0±56.16** |
| Riyadh-1 | 600.3±8.74 | 527.0±7.00 | 558.3±8.30 | 496.2±10.25 | **545.5±40.92** |
| Yecora | 509.6±17.53 | 438.7±12.88 | 461.4±12.37 | 396.0±8.37 | **451.4±44.17** |
| **Mean** | **520.9±41.87** | **424.2±53.2** | **484.4±38.03** | **404.6±44.08** | |
| **$LSD_{g0.05\%}$** | **8.366** | | | | |
| **$LSD_{e0.05\%}$** | **3.104** | | | | |
| **$LSD_{g\times e0.05\%}$** | **16.35** | | | | |
| | | **Thousand grain weight (g)** | | | |
| ACS-1346 | 45.19±1.67 | 39.81±1.50 | 42.43±0.75 | 38.23±1.27 | **41.42±2.99** |
| ACS-1372 | 45.93±1.63 | 40.10±0.26 | 41.78±1.14 | 34.11±2.31 | **40.48±4.63** |
| ACS-1400 | 45.97±1.73 | 41.41±1.20 | 43.76±1.60 | 39.76±1.58 | **42.73±2.79** |
| ACS-1422 | 48.50±1.02 | 43.34±1.13 | 45.38±1.89 | 39.54±0.46 | **44.19±3.55** |
| ACS-1444 | 46.68±1.07 | 38.42±0.91 | 46.06±1.73 | 39.19±0.83 | **42.59±4.10** |
| ACS-1454 | 45.80±1.06 | 41.67±0.77 | 43.02±1.10 | 39.66±0.46 | **42.54±2.45** |
| ACS-1456 | 47.15±1.03 | 36.77±0.64 | 45.51±1.36 | 37.15±0.66 | **41.65±5.00** |
| ACS-1464 | 47.48±1.19 | 40.26±0.89 | 42.08±2.46 | 41.25±0.36 | **42.77±3.17** |
| Asier-1 | 45.17±1.73 | 38.89±1.32 | 41.65±0.78 | 38.80±1.11 | **41.13±2.93** |
| LR-12 | 47.29±1.52 | 41.00±1.69 | 44.93±0.75 | 38.32±1.49 | **42.88±3.81** |
| LR-599 | 43.53±0.92 | 37.36±1.72 | 39.67±0.67 | 34.83±0.93 | **38.85±3.48** |
| LR-920 | 49.18±0.84 | 40.34±1.64 | 44.96±1.82 | 42.11±1.37 | **44.15±3.71** |
| Maeaa | 46.39±1.22 | 40.83±1.59 | 43.67±1.58 | 37.54±0.88 | **42.11±3.62** |
| Riyadh-1 | 47.45±1.13 | 42.24±1.43 | 44.41±1.59 | 38.56±1.56 | **43.17±3.60** |
| Yecora | 46.92±0.67 | 41.44±1.05 | 44.15±1.47 | 37.65±1.48 | **42.538±3.72** |
| **Mean** | **46.58±1.73** | **40.26±2.04** | **43.56±2.11** | **38.45±2.29** | |
| **$LSD_{g0.05\%}$** | **1.051** | | | | |
| **$LSD_{e0.05\%}$** | **0.449** | | | | |
| **$LSD_{g\times e0.05\%}$** | **2.062** | | | | |

Values are presented as mean ± standard deviation. LSDg, least significant difference for comparing genotype means at the 5% probability level; LSDe, least significant difference for comparing environment means at the 5% probability level; LSDg×e, least significant difference for comparing genotype by environment interaction means at the 5% probability level.

**Table S2.** Mean performance of fifteen bread wheat genotypes for plant height and spike length across two contrasting locations in Saudi Arabia (Riyadh and Hail) during the 2023 and 2024 growing seasons.

| Genotype | Hail-23 | Riyadh-23 | Hail-24 | Riyadh-24 | Mean |
|---|---|---|---|---|---|
| | | | **Plant height (cm)** | | |
| ACS-1346 | 106.6±2.92 | 100.8±2.55 | 104.1±3.48 | 94.40±1.44 | **101.5±5.31** |
| ACS-1372 | 104.8±1.65 | 101.2±1.24 | 102.5±2.32 | 97.12±0.97 | **101.4±3.21** |
| ACS-1400 | 102.0±3.21 | 95.59±1.23 | 98.14±2.07 | 91.08±1.66 | **96.70±4.54** |
| ACS-1422 | 102.7±3.03 | 94.80±4.30 | 99.54±3.71 | 89.75±2.95 | **96.71±5.95** |
| ACS-1444 | 108.0±1.76 | 94.82±1.27 | 106.0±3.65 | 96.76±1.65 | **101.4±6.24** |
| ACS-1454 | 98.12±2.82 | 92.33±2.52 | 95.07±1.85 | 88.77±1.37 | **93.57±4.07** |
| ACS-1456 | 109.4±2.11 | 90.79±1.58 | 107.8±2.71 | 89.26±1.28 | **99.31±9.88** |
| ACS-1464 | 100.1±4.15 | 83.23±2.04 | 89.88±0.83 | 84.32±2.19 | **89.38±7.32** |
| Asier-1 | 98.38±4.11 | 92.29±2.06 | 98.49±5.64 | 92.68±1.53 | **95.46±4.44** |
| LR-12 | 127.5±1.34 | 116.4±1.65 | 122.9±1.87 | 103.9±2.84 | **117.7±9.42** |
| LR-599 | 96.86±1.62 | 92.46±1.61 | 95.22±0.70 | 89.14±1.65 | **93.42±3.30** |
| LR-920 | 115.4±2.51 | 94.96±2.72 | 103.8±2.11 | 96.22±2.91 | **102.6±8.77** |
| Maeaa | 115.8±1.40 | 103.6±1.65 | 110.8±1.05 | 98.33±3.51 | **107.1±7.21** |
| Riyadh-1 | 100.0±2.65 | 96.33±1.15 | 97.00±4.36 | 88.00±4.36 | **95.33±5.48** |
| Yecora | 87.33±2.52 | 78.33±2.08 | 86.97±2.37 | 77.40±1.44 | **82.51±5.19** |
| **Mean** | **104.9±9.65** | **95.19±7.68** | **101.2±8.93** | **91.81±6.54** | |
| **$LSD_{g0.05\%}$** | **2.026** | | | | |
| **$LSD_{e0.05\%}$** | **1.405** | | | | |
| **$LSD_{g\times e0.05\%}$** | **4.075** | | | | |
| | | | **Spike length (cm)** | | |
| ACS-1346 | 13.74±0.46 | 12.93±0.13 | 13.49±0.88 | 12.53±0.92 | **13.17±0.76** |
| ACS-1372 | 13.00±0.39 | 12.00±0.22 | 12.67±0.58 | 11.60±0.72 | **12.32±0.70** |
| ACS-1400 | 13.87±0.23 | 12.73±0.31 | 13.00±1.00 | 12.13±0.73 | **12.93±0.86** |
| ACS-1422 | 13.00±1.00 | 11.87±0.71 | 12.87±1.21 | 12.20±0.87 | **12.48±0.96** |
| ACS-1444 | 14.47±0.92 | 11.54±0.41 | 14.27±1.30 | 11.91±0.38 | **13.05±1.56** |
| ACS-1454 | 14.80±0.72 | 12.98±0.80 | 13.69±1.10 | 13.13±0.64 | **13.65±1.03** |
| ACS-1456 | 13.33±0.58 | 11.17±1.26 | 13.17±0.76 | 10.52±0.63 | **12.05±1.47** |
| ACS-1464 | 14.07±0.90 | 10.32±0.56 | 11.77±0.40 | 10.70±0.46 | **11.71±1.61** |
| Asier-1 | 13.45±0.79 | 11.50±1.32 | 13.14±0.80 | 11.80±1.11 | **12.47±1.24** |
| LR-12 | 13.50±0.50 | 11.77±0.68 | 13.37±0.64 | 11.33±0.70 | **12.49±1.14** |
| LR-599 | 13.67±1.15 | 11.46±0.44 | 12.60±0.69 | 10.90±0.36 | **12.16±1.28** |
| LR-920 | 12.43±0.52 | 10.27±0.83 | 12.42±0.73 | 10.78±0.78 | **11.48±1.18** |
| Maeaa | 13.05±0.93 | 11.89±0.18 | 12.74±0.65 | 11.67±0.61 | **12.34±0.82** |
| Riyadh-1 | 13.92±0.89 | 12.56±0.51 | 13.59±0.52 | 12.09±0.61 | **13.04±0.96** |
| Yecora | 11.71±0.51 | 10.72±0.96 | 11.11±0.85 | 10.28±0.83 | **10.95±0.88** |
| **Mean** | **13.47±0.97** | **11.71±1.03** | **12.93±1.03** | **11.57±0.99** | |
| **$LSD_{g0.05\%}$** | **0.617** | | | | |
| **$LSD_{e0.05\%}$** | **0.430** | | | | |
| **$LSD_{g\times e0.05\%}$** | **1.207** | | | | |

Values are presented as mean ± standard deviation. LSDg, least significant difference for comparing genotype means at the 5% probability level; LSDe, least significant difference for comparing environment means at the 5% probability level; LSDg×e, least significant difference for comparing genotype by environment interaction means at the 5% probability level.

**Table S3**. Mean performance of fifteen bread wheat genotypes for days to heading and days to physiological maturity across two contrasting locations in Saudi Arabia (Riyadh and Hail) during the 2023 and 2024 growing seasons.

| Genotype | Hail-23 | Riyadh-23 | Hail-24 | Riyadh-24 | Mean |
|---|---|---|---|---|---|
| | | | **Days to heading** | | |
| ACS-1346 | 92.58±0.72 | 84.08±1.48 | 90.59±1.23 | 81.48±2.82 | **87.18±4.98** |
| ACS-1372 | 89.96±1.70 | 84.69±2.15 | 87.49±1.79 | 82.80±0.35 | **86.24±3.17** |
| ACS-1400 | 87.82±2.04 | 81.09±0.98 | 85.90±2.16 | 76.81±1.29 | **82.91±4.71** |
| ACS-1422 | 88.53±1.75 | 76.93±1.11 | 85.23±1.33 | 72.63±1.59 | **80.83±6.75** |
| ACS-1444 | 86.89±6.27 | 78.53±1.76 | 86.51±1.79 | 79.21±1.41 | **82.79±5.05** |
| ACS-1454 | 84.96±2.58 | 77.46±2.28 | 80.82±1.64 | 75.37±1.48 | **79.65±4.17** |
| ACS-1456 | 89.93±1.11 | 80.12±1.18 | 89.23±2.54 | 80.08±1.12 | **84.84±5.14** |
| ACS-1464 | 90.88±1.64 | 81.75±1.30 | 83.52±1.30 | 82.75±0.66 | **84.73±3.92** |
| Asier-1 | 89.96±1.06 | 76.18±2.04 | 87.59±1.22 | 77.15±1.99 | **82.72±6.55** |
| LR-12 | 94.49±0.88 | 86.82±1.28 | 92.44±1.71 | 85.42±1.46 | **89.79±4.11** |
| LR-599 | 93.88±1.18 | 81.63±1.59 | 90.38±1.07 | 80.62±1.61 | **86.63±6.02** |
| LR-920 | 93.15±0.79 | 85.49±0.88 | 90.71±1.45 | 86.52±0.83 | **88.97±3.36** |
| Maeaa | 93.27±1.42 | 83.32±2.15 | 91.45±1.58 | 82.75±0.66 | **87.70±5.09** |
| Riyadh-1 | 88.56±1.25 | 81.05±1.93 | 85.76±2.15 | 79.62±1.61 | **83.75±4.04** |
| Yecora | 86.23±1.37 | 79.13±0.81 | 81.20±1.31 | 76.97±1.70 | **80.88±3.76** |
| **Mean** | **90.08±3.38** | **81.22±2.82** | **87.26±3.78** | **80.01±3.91** | |
| **$LSD_{g0.05\%}$** | **1.340** | | | | |
| **$LSD_{e0.05\%}$** | **1.094** | | | | |
| **$LSD_{g\times e0.05\%}$** | **2.737** | | | | |
| | | | **Days to maturity** | | |
| ACS-1346 | 137.4±1.42 | 128.8±2.22 | 135.5±1.82 | 124.5±1.79 | **131.5±5.64** |
| ACS-1372 | 132.8±1.40 | 127.4±1.42 | 130.4±1.42 | 125.8±1.33 | **129.1±3.05** |
| ACS-1400 | 130.5±1.38 | 124.3±0.63 | 127.0±1.94 | 120.8±2.05 | **125.6±3.97** |
| ACS-1422 | 132.5±2.28 | 123.7±1.44 | 128.8±1.30 | 117.8±1.36 | **125.7±5.95** |
| ACS-1444 | 131.5±1.31 | 123.8±1.28 | 131.1±2.00 | 125.1±1.69 | **127.9±3.86** |
| ACS-1454 | 129.6±0.70 | 121.7±1.44 | 126.1±2.44 | 118.8±1.40 | **124.1±4.54** |
| ACS-1456 | 131.4±1.07 | 120.6±1.96 | 130.8±1.94 | 121.0±2.00 | **125.9±5.60** |
| ACS-1464 | 132.6±1.23 | 121.1±1.09 | 124.8±0.73 | 122.9±1.52 | **125.3±4.70** |
| Asier-1 | 132.8±1.91 | 122.2±2.19 | 131.8±1.60 | 123.1±1.70 | **127.5±5.33** |
| LR-12 | 135.1±1.52 | 128.6±1.59 | 135.0±1.06 | 127.4±1.07 | **131.5±3.89** |
| LR-599 | 138.4±0.72 | 125.5±1.85 | 134.9±1.49 | 125.3±1.41 | **131.0±6.14** |
| LR-920 | 137.2±2.28 | 123.8±1.34 | 134.6±2.17 | 125.1±0.84 | **130.2±6.26** |
| Maeaa | 133.7±1.45 | 129.6±1.27 | 133.3±3.53 | 128.1±0.93 | **131.2±3.06** |
| Riyadh-1 | 131.2±1.73 | 120.8±1.43 | 125.8±1.75 | 118.5±1.76 | **124.1±5.29** |
| Yecora | 134.2±1.23 | 126.0±1.06 | 132.8±1.56 | 124.4±1.42 | **129.3±4.53** |
| **Mean** | **133.4±2.86** | **124.5±3.21** | **130.8±3.86** | **123.2±3.38** | |
| **$LSD_{g0.05\%}$** | **1.327** | | | | |
| **$LSD_{e0.05\%}$** | **0.651** | | | | |
| **$LSD_{g\times e0.05\%}$** | **2.616** | | | | |

Values are presented as mean ± standard deviation. LSDg, least significant difference for comparing genotype means at the 5% probability level; LSDe, least significant difference for comparing environment means at the 5% probability level; LSDg×e, least significant difference for comparing genotype by environment interaction means at the 5% probability level.

**Table S4.** Pedigree, classification, origin, and year of release of the fifteen wheat genotypes evaluated in the present study.

| Genotype | Classification | Pedigree | Origin | Year of release |
|---|---|---|---|---|
| ACS_1456 | Advanced line | ACSAD 1123 /4/ SAAR/3/C80.1/3BATAVIA//2WBLL1<br>ACS-W-11166 (2012) - 21IZ-11IZ-21IZ | ACSAD | - |
| ACS-1346 | Advanced line | ACSAD1133/3/TOBA97/BAV92//MILAN/KAUZ<br>ACS-W-10591 (2008) - 23IZ-11IZ-11IZ-01IZ | ACSAD | - |
| ACS-1372 | Advanced line | ACSAD 1069/4/KAUZ/LUCO-M//PVN/STAR/3/FOW-1<br>ACS-W-10766 (2009) - 31Z-11IZ-21IZ-01IZ | ACSAD | - |
| ACS-1400 | Advanced line | WIFAK /3/ WHEAR/KUKUNA/WHEAR<br>ACS-W-11127 (2012) - 21IZ-21IZ-11IZ | ACSAD | - |
| ACS-1422 | Advanced line | ACSAD 1115 / GEMMEIZA9<br>ACS-W-11097 (2012) - 21IZ-21IZ-11IZ | ACSAD | - |
| ACS-1444 | Advanced line | ACSAD 1178/6/SHARP/3/PRL/SARA/TSI/VEE#5/5/VEE/LIRA/BOW<br>ACS-W-11130 (2012) - 91Z-21IZ-21IZ | ACSAD | - |
| ACS-1454 | Advanced line | ACSAD 1198 // ND643/2*WBLL1<br>ACS-W-11151 (2012) - 23IZ-21IZ-31IZ | ACSAD | - |
| ACS-1464 | Advanced line | ACSAD 1147 /4/ SAAR/3/C80.1/3BATAVIA//2WBLL1<br>ACS-W-11170 (2012) - 21IZ-31IZ-31IZ | ACSAD | - |
| LR-12 | Landrace | Saudi landrace was collected from the Tumair Region in the year 2010 | Saudi Arabia | - |
| LR-599 | Landrace | Saudi landrace was registered in the Saudi Genebank in 2011 | Saudi Arabia | - |
| LR-920 | Landrace | Saudi landrace was collected from the Asir Region in the year 2013 | Saudi Arabia | - |
| Asier-1 | Local variety | Local variety | Saudi Arabia | 2023 |
| Reyadh-1 | Local variety | Local variety | Saudi Arabia | 2023 |
| Maeaa | Local variety | Local variety | Saudi Arabia | 2023 |
| Yecora Rojo | imported cultivar | Ciano 67 // Sonora 64 / Klein Rendidor / 3 / II-8156 | CIMMYT | 1970 |

**Table S5.** Monthly climatic data at Riyadh and Hail during the 2022–2023 and 2023–2024 growing seasons.

| Location | Month | Minimum temperature (°C) | Maximum temperature (°C) | Humidity (%) | Precipitation (mm) |
|---|---|---|---|---|---|
| | | **Season 2022-2023** | | | |
| **Riyadh** | November | 11.0 | 29.6 | 38.0 | 15.5 |
| | December | 5.40 | 26.0 | 56.0 | 10.5 |
| | January | 10.2 | 17.8 | 73.0 | 13.9 |
| | February | 12.0 | 23.6 | 42.0 | 12.9 |
| | March | 16.3 | 28.4 | 40.0 | 16.6 |
| | April | 18.7 | 30.6 | 39.0 | 20.8 |
| | May | 25.5 | 38.2 | 22.0 | 8.79 |
| | | **Season 2023-2024** | | | |
| | November | 17.6 | 27.9 | 47.0 | 10.4 |
| | December | 11.6 | 24.3 | 38.0 | 8.2 |
| | January | 12.9 | 29.7 | 39.0 | 10.0 |
| | February | 10.4 | 33.7 | 38.0 | 16.0 |
| | March | 13.6 | 33.0 | 32.0 | 11.2 |
| | April | 18.2 | 37.1 | 30.0 | 15.2 |
| | May | 19.0 | 44.7 | 19.0 | 5.80 |
| | | **Season 2022-2023** | | | |
| **Hail** | November | 8.60 | 26.6 | 60.0 | 22.6 |
| | December | 1.40 | 20.4 | 66.0 | 15.7 |
| | January | 5.90 | 15.5 | 72.0 | 25.4 |
| | February | 5.60 | 18.1 | 42.0 | 27.6 |
| | March | 10.7 | 22.8 | 47.0 | 30.7 |
| | April | 13.7 | 26.5 | 47.0 | 48.8 |
| | May | 19.8 | 33.3 | 26.0 | 25.9 |
| | | **Season 2023-2024** | | | |
| | November | 13.0 | 24.7 | 60.0 | 12.5 |
| | December | 8.60 | 20.5 | 56.0 | 6.7 |
| | January | 3.70 | 25.0 | 58.0 | 20.4 |
| | February | 5.10 | 26.8 | 47.0 | 25.2 |
| | March | 6.50 | 29.2 | 44.0 | 41.5 |
| | April | 14.4 | 33.3 | 36.0 | 42.6 |
| | May | 15.6 | 39.4 | 24.0 | 21.6 |
